# Snake Electrocution on Power Distribution Infrastructure in South Korea: Species Composition, Spatiotemporal Patterns, and Spatial Risk Modelling

**DOI:** 10.3390/biology15211958

**Published:** 2026-11-01

**Authors:** Jeongwoo Yoo, Jung-Hyun Lee

**Affiliations:** Research Center for Endangered Species, National Institute of Ecology, 23 Gowol-gil, Yeongyang-eup, Yeongyang-gun 36531, Republic of Korea; wildlife3028@nie.re.kr

**Keywords:** electrocution, *Elaphe schrenckii*, endangered species, forest–farmland mosaic, human–wildlife conflict, power distribution network, risk mapping, snake

## Abstract

Snake electrocution on power distribution infrastructure remains poorly quantified. We analysed Korea Electric Power Corporation records from January 2024 to June 2025, including three additional records from early July 2025. Among 449 recorded incidents, photographs identified 324 individuals of 6 species from 323 incidents. The nationally endangered Amur ratsnake (*Elaphe schrenckii*) accounted for 202 individuals (62.3%). Spatial analyses used all 426 georeferenced records. Incidents peaked in June and occurred during daylight more often than expected from day length. The rate in Daegu–Gyeongbuk was 4.27 times that in the rest of the country (95% CI: 3.53–5.17). A case–control logistic model used 10 environmental variables, 189 case records, and 7341 control poles. Higher odds were associated with joint proximity to forest and farmland, sparse pole networks, and proximity to settlements and watercourses. External validation in an independent provincial case–control sample yielded an AUC of 0.683 (95% CI: 0.586–0.771). Across the full provincial inventory, the highest-ranked 30% of poles included 44.8% of incident poles (95% CI: 26.4–64.3%), compared with 30% expected under random selection. Predictive performance was modest and requires further evaluation before operational use.

## Simple Summary

Electricity distribution lines cross farmland and forests worldwide, and climbing animals can be killed when they contact energised equipment. Birds have been studied extensively, but snake electrocution remains poorly documented, particularly when it does not interrupt supply. We examined records from the Korean national electricity utility covering an 18-month extraction window and three additional incidents in early July 2025. Among 449 recorded incidents, photographs allowed 324 snakes to be identified to species. Almost two-thirds were Amur ratsnakes, which are nationally endangered in the Republic of Korea. Photographs also showed features suggestive of possible recent feeding or gravidity, and one pair with genital association consistent with mating. Incidents peaked in June and occurred during daylight more often than expected from day length. Regional rates differed even after accounting for pole numbers. We developed a model to rank poles by their association with recorded incidents. Higher-ranked poles tended to have both forest and farmland nearby and to occur in sparse parts of the network. External validation indicated modest predictive performance, supporting further evaluation of the ranking as a possible aid to field assessment rather than as a tool for selecting poles for treatment.

## 1. Introduction

Electricity supply infrastructure is a foundation of human society and overlaps extensively with wildlife habitat. Utility poles are artificial vertical structures distributed regularly across open landscapes—birds use them for resting, perching, and nesting, whereas climbing mammals and reptiles use them as movement routes or objects of exploration [1]. When an animal simultaneously contacts an energised conductor and a grounded structure, or two conductors at different electrical potentials, the resulting short circuit can kill the animal and interrupt supply. Where contact is severe enough to generate a fault, these two outcomes coincide: the same event is a source of anthropogenic mortality for wildlife and an operational fault for the utility [2]. Contact that does not generate a fault may still kill the animal, and such events do not enter utility records at all. The alignment, therefore, holds for the outage-confirmed subset rather than for snake mortality on power infrastructure as a whole. This dual character has been recognised mainly through work on birds, particularly raptors, for which electrocution can contribute to population decline and for which methods now exist to identify hazardous poles and to prioritise retrofitting and insulation [3,4]. Animal-related faults recur in distribution-system records more generally [5,6], and risk analyses and mitigation technologies have been developed for pole use by birds and equipment damage by rodents [7].

Snakes have received far less attention, although their morphology and climbing ability can expose them to distribution equipment. Snakes able to climb vertical structures such as poles can reach wires, insulators, and transformers, and an elongated body can bridge a grounded structure and a conductor, or two conductors, more readily than a compact one [8]. On Guam, the introduced brown treesnake (*Boiga irregularis*) caused power-facility incidents repeatedly over many years, with estimated losses exceeding USD 4.5 million annually, and its climbing ability and long body were identified as the traits enabling access to and electrical contact with infrastructure [8]. The scale of that problem was recognised early precisely because the outages themselves were informative: Fritts, Scott, and Savidge used the timing of electrical outages to infer the activity of the species on Guam, an early use of utility fault records as a record of snake behaviour [9]. Recent work has shown that non-avian electrocution, including reptile and snake electrocution, is more widespread than previously recognised: among 120 non-avian electrocution incidents reported by utility workers in Iran, snakes accounted for 19.2% [10]. Nevertheless, spatially explicit, species-level analyses linking snake electrocution to landscape characteristics remain scarce. A central reason is detection: a dead snake on a pole is rarely found by a surveyor, and in practice becomes a record only when the contact is severe enough to interrupt supply and bring a repair crew to the site. Mortality that does not cause a fault is, therefore, largely invisible, and quantitative analyses of which species are killed, when, and at which poles are correspondingly scarce.

The Republic of Korea provides an opportunity to address this gap because snake-related faults are logged nationally. Among the species identified in the present study, the Amur ratsnake (*Elaphe schrenckii*) uses forest, farmland, settlements, and watercourses in mosaic landscapes and may, therefore, encounter power infrastructure in rural areas [11]. It is one of the largest snakes in the country, reaching 200 cm in total length, and is designated a Class II endangered species under the Wildlife Protection and Management Act, administered by the Ministry of Climate, Energy, and Environment (formerly the Ministry of Environment). Snakes have life histories that are sensitive to increases in adult mortality because maturity is late and annual reproductive output is limited [12,13], so a persistent, infrastructure-linked source of adult mortality is of conservation concern even when the number of recorded individuals is modest. At the same time, each of these deaths is an outage for the utility and its customers, which makes utility-pole electrocution a two-sided conflict rather than a purely ecological problem. Despite this, no study has yet quantified snake electrocution in Korea at the species level or characterised the environmental setting of the poles at which it occurs.

Where electrocution occurs on a distribution network should not be spatially random, because snakes are not randomly distributed across landscapes. Large colubrids in temperate agricultural regions concentrate their activity where structurally contrasting habitats meet. Forest edges adjoining farmland support high densities of small rodents and ground-nesting birds, the principal prey of these species [14], and such ecotones are zones of high prey density and concentrated animal movement [15]—riparian vegetation functions as a movement corridor [11]. Studies of snake road mortality in Korea show that encounters with linear infrastructure are concentrated where these habitats abut the structure rather than being distributed in proportion to its extent [16,17]. Poles standing in such settings should, therefore, be encountered, and climbed, more often than poles within continuous forest, in open farmland distant from cover, or in dense urban networks. A second, non-exclusive expectation is thermal: snakes exploit artificial structures for thermoregulation [18,19], so poles receiving greater solar radiation might be used preferentially.

Our objectives were, therefore, threefold. First, we quantified the species composition of outage-confirmed electrocution, in order to examine whether the recorded pattern was consistent with the ecological and morphological traits of the species affected—in particular with the wide-ranging movement of active foragers [20] and with the behavioural repertoire and body form required to climb a smooth cylindrical structure [21,22]. Because the abundance of each species in the study region is unknown, this objective is descriptive and does not allow species-specific electrocution risk to be estimated. Second, we described the seasonal and diel distribution of recorded incidents. Third, we examined whether recorded electrocution at a pole was associated with joint proximity to forest and farmland, with proximity to watercourses, and with the configuration of the pole network, using a case–control design. We did not measure a mapped forest–farmland boundary, so this is a test of the landscape setting in which the two cover types are both close to the pole rather than of an edge effect as such, and we evaluated the spatial transferability of the resulting risk model and its value as a first-pass screening tool for prioritising poles for field assessment.

## 2. Materials and Methods

### 2.1. Electrocution Records

#### 2.1.1. Data Collection

We used incident-handling records managed by the Korea Electric Power Corporation (KEPCO) and retained the cases classified as snake contact. The register was extracted for the period 1 January 2024 to 30 June 2025, a window of 18 months. Three further incidents dated 1–4 July 2025 were also present in the extract and are retained throughout except in the monthly frequency test, which is restricted to the complete extraction window (Section 2.3). All three carry coordinates and a time, so all three enter the regional rate comparison (one each in Chungbuk, Daejeon–Sejong–Chungnam, and Gyeongnam) and the diel analysis, and the two identified to species enter the monthly figure but not the frequency tests in Table S7. None falls in the model training region, so none enters the case–control model. A total of 450 snake-contact incidents were listed in the extract. Two entries were identical in every recorded field—regional headquarters, branch office, date, time to the second, address, pole number, and computerised pole identifier—and in both the species field was blank—we treated them as one incident entered twice and removed one of the pair, leaving 449 incidents. A second pair of entries also shared a pole and a logging time but recorded different species, each determined from its own carcass photograph—these were retained as two records (Section 3.1). The earliest incident was on 9 January 2024 and the latest on 4 July 2025—435 (96.9%) were momentary interruptions and 14 (3.1%) were sustained interruptions.

Of the 449 incidents, 323 were accompanied by carcass photographs taken by the responding field crew from which the species could be determined. The remaining 126 either lacked photographs (93) or had photographs from which the species could not be determined (33). Examples of each outcome are shown in Figures S3 and S4. Two steppe ratsnakes were electrocuted in a single incident, so the 323 species-identified incidents correspond to 324 individuals—incidents and individuals are distinguished throughout.

Pole coordinates are recorded from the pole identifier rather than from the photograph and were, therefore, available for most incidents irrespective of species identification: 426 of the 449 incidents (94.9%) carried coordinates, including 103 of the 126 incidents without species identification. We, therefore, define the following analysis sets, which are used consistently below and summarised in Figure S1:

- Set A—All 449 incidents. Used for the regional comparison, which requires only the regional headquarters field.
- Set B—The 426 incidents with coordinates. Used for the spatial analyses.
- Set C—Set B excluding those of the seven incidents whose field notes record a different or uncertain cause (four bird contact, two tree contact, and one “presumed snake contact”) that carry coordinates. Three of the seven do. Set C, therefore, contains 423 incidents.
- Set D—Set C excluding two further incidents whose remarks field indicates a different cause (“suspected roadkill”; “not a utility pole, presumed substation equipment”), both of which carry coordinates—421 incidents. In the training region the corresponding case sets are 187 and 186 records.
- Set E—The 323 species-identified incidents. Used for the species composition analysis, for which a usable photograph is a necessary condition, and reported as a sensitivity analysis elsewhere.

The spatial analyses below use Set B as the primary set, with Sets C and D as sensitivity analyses and Set E as a further sensitivity analysis.

Because detection depends on a fault being generated and a crew being dispatched, the records describe electrocution events that caused an outage rather than the full extent of snake contact with power infrastructure. The register does not record whether the procedures for coding an outage cause as snake contact remained unchanged across the study period, and we were not able to establish this independently. We, therefore, cannot distinguish how far the higher number of June incidents in 2025 than in 2024 reflects a change in incidence rather than a change in reporting or classification practice. We return to this limitation in Section 4.3.

Among the ten regions with at least five incidents, the proportion for which the species could be identified varied from 44.2% to 100% (Table S2; the two incidents recorded in Jeju, neither identified, are listed but not included in this range or the test), and this variation was greater than expected by chance (χ^2^ = 35.4, df = 9, *p* = 5.1 × 10^−5^, regions with ≥5 incidents). Restricting the spatial analyses to species-identified incidents would, therefore, have introduced spatially structured missingness. This is the reason the spatial analyses use Set B rather than Set E.

#### 2.1.2. Species Identification and Condition

Species were identified from the carcass photographs by a herpetologist (J.-H. Lee). Photographs showing the whole animal were used, and identification was based on the principal diagnostic keys for the regional snake fauna—overall body size, body form, and the dorsal colour pattern and the presence and arrangement of blotches or bands—with reference to a regional field guide [23]. Each of the species recorded possesses distinctive external characters, so a photograph showing the whole animal was generally sufficient. Scientific and English names follow the National Species List of Korea [24]. Where the animal was obscured by vegetation or by the structure, damaged by the fault, photographed from a distance or at an angle that concealed these characters, or recorded only at night, the incident was classified as not identifiable and assigned to the 126 unidentified incidents rather than to a best guess. Representative photographs are provided for identifiable carcasses (Figure S3) and for each of these failure modes (Figure S4).

No operational criterion was available for separating juveniles from adults in these photographs: the images contain no scale object, and species-specific length thresholds for maturity could not be applied to an animal whose length was estimated against infrastructure in the same frame. Age class is, therefore, not assigned and is not reported in this paper.

Condition was assigned from the photographs on two grounds together—the shape of the abdomen and the time of year. *Elaphe schrenckii*, *E. dione*, and *Rhabdophis tigrinus* in this region typically mate in April and May and lay in July and August. On that basis, an abdomen evenly distended along its whole length was interpreted as consistent with possible gravidity, and a single discrete swelling in part of the abdomen as consistent with possible recent feeding. One individual, in which an egg was partly extruded in the photograph, is the only case in which gravidity is directly evidenced. One pair showed visible genital association interpreted as consistent with mating—the photographs were taken after the incident, so we do not infer from them what the animals were doing at the moment of contact. Individuals showing none of these features were recorded as having no notable features. These are assessments of photographic appearance and were not independently confirmed. The photographic basis for each of the three categories is illustrated in Figure S5.

These categories rest on external appearance without dissection or independent confirmation, and the interpretation in Section 4.1 is qualified accordingly.

### 2.2. Spatial Risk Modelling

#### 2.2.1. Study Regions

The modelling region was Daegu Metropolitan City and Gyeongsangbuk-do (hereafter Daegu–Gyeongbuk; the jurisdictions of the KEPCO Daegu and Gyeongbuk regional headquarters), where electrocution was most concentrated (Figure 1a). The region lies between 35° and 37° N and 127° and 130° E and comprises a temperate mosaic of oak- and pine-dominated montane forest, paddy and dry field agriculture, riparian corridors, and urban and peri-urban areas. It contains 761,520 utility poles. Of the 212 snake-contact incidents recorded in the region, 189 carried coordinates and were located at 184 poles—148 of the 212 were identified to species.

Chungcheongbuk-do (hereafter Chungbuk; the jurisdiction of the KEPCO Chungbuk regional headquarters) was selected for external validation because it is, like Daegu–Gyeongbuk, a landlocked inland province dominated by an agriculture–forest mosaic, so landscape structure is comparable, a sufficient number of incidents was available, and it is administratively and geographically separate from the modelling region, ensuring data independence. Chungbuk contains 264,036 utility poles. All 32 snake-contact incidents recorded in the province carried coordinates and were located at 31 poles—26 were identified to species.

#### 2.2.2. Environmental Variables

Ten environmental variables were extracted at each pole location (Table 1). The four domains they represent—habitat structure, network configuration, terrain, and energy conditions—follow the expectations set out in the Introduction. All ten variables were entered simultaneously and retained—none was selected, dropped, or transformed on the strength of its association with the outcome. All spatial data were projected to the Korea 2000/Central Belt 2010 coordinate system (EPSG:5186). The incident register is distributed in the Korea 2000/Central Belt coordinate system with a false northing of 500,000 m and was transformed to EPSG:5186 before use.

Land-cover variables were derived from the medium-classification (Level 2) land-cover map distributed by the Environmental Geographic Information Service of the Ministry of Climate, Energy, and Environment (https://egis.me.go.kr, accessed on 21 May 2026), which is compiled at a nominal scale of 1:25,000 with a spatial resolution of 5 m and 22 classes—the 2024 edition was used. Forest comprised classes 310, 320, and 330 (broadleaf, coniferous, and mixed forest), farmland comprised classes 210–250 (paddy, dry field, protected cultivation, orchard, and other cultivation), and settlement comprised classes 110–160 (residential, industrial, commercial, cultural and sports facilities, transport, and public facilities). The same class definitions were applied in both study regions. Watercourses were taken from the national stream network layer.

Distance variables were computed as Euclidean distances from each pole to the nearest polygon or line of the relevant class. The distances to forest, to farmland, and to settlement were all extracted from the same polygon layer under the class definitions given above. Recomputing them from the archived polygons reproduces the distances to farmland and to settlement exactly and the distance to forest to within 0.05 m, the precision at which it is stored. This single provenance matters because FF_PROX, defined next, combines two of them. Joint proximity to forest and farmland (FF_PROX) was defined as the larger of the distance to forest and the distance to farmland, which is the radius of the smallest circle centred on the pole that contains both land-cover classes. Smaller values, therefore, indicate a pole that has both land-cover types nearby. The index quantifies joint proximity to the two classes, and it is not the distance to a mapped boundary that the two classes share. A pole can have forest and farmland within a short distance of it while the two are separated by a road or by built-up land, and in that case FF_PROX is small, although no forest–farmland boundary lies nearby. The index is, therefore, a proxy for the landscape setting in which forest and farmland adjoin, not a direct measurement of an edge. Extracting the shared boundary itself from the land-cover map and measuring distance to it remains a direction for further work (Section 4.3). FF_PROX replaces the separate distances to forest and to farmland, with which it is collinear (r = 0.92 and 0.83 on the transformed scale; entering all three together raised the maximum variance inflation factor to 18.2). It is a deterministic function of those two distances and was not chosen from among alternative formulations on the basis of its association with the outcome. Forest cover was the proportion of forest within a 200 m radius. Pole density was the number of poles, including the pole itself, within a 1 km × 1 km window centred on the pole; because the window area is 1 km^2^, this count is also the density per km^2^.

The normalised difference vegetation index (NDVI) was derived from Sentinel-2 Level-2A surface-reflectance imagery as (NIR − Red)/(NIR + Red) using band 8 (10 m) and band 4 (10 m). A composite was produced on the Google Earth Engine platform [25] from the COPERNICUS/S2_SR_HARMONIZED collection over 1 January to 31 December 2024, retaining scenes with a CLOUDY_PIXEL_PERCENTAGE below 20 and taking the arithmetic mean of the per-scene NDVI images. The composite was exported at 10 m in EPSG:4326, and values were extracted at the pole location by nearest-neighbour sampling. Three properties of this composite should be noted. The cloud criterion is applied at the level of the scene and not of the pixel, no per-pixel quality-band masking was used, and the compositing statistic is the mean rather than the median, so residual cloud within retained scenes is averaged into the composite and may have biased NDVI at affected locations. The window is the full calendar year rather than the growing season, so the variable is an annual mean and not a peak-greenness measure. NDVI was not significant in the fitted model (Section 3.5), and the land-cover-derived habitat variables, which carry the principal results, are independent of it. Elevation, slope, and aspect were derived from the 30 m national digital elevation model published by the National Geographic Information Institute of the Republic of Korea, with slope and aspect computed by Horn’s method.

Annual plane-of-array solar radiation (SOLAR, kWh m^−2^ yr^−1^) was computed with pvlib-python 0.16.1 [26], using the isotropic transposition model with a ground albedo of 0.25. The modelled surface was a plane with the tilt and azimuth of the terrain at the pole, taken from the DEM-derived slope and aspect. Clear-sky irradiance was generated with the Ineichen model for a single reference location in each study region (128.5° E, 36.2° N for Daegu–Gyeongbuk; 127.9° E, 36.8° N for Chungbuk) at solar noon on the fifteenth day of each month and converted to a monthly total by multiplying by eight hours per day and by the number of days in the month. The twelve monthly totals were then summed. This is a coarse representation: it uses twelve instantaneous clear-sky values rather than a continuous time series, assumes a single solar geometry across each region, and takes no account of cloud cover or of shading by surrounding terrain. SOLAR should, therefore, be read as a relative index of terrain-driven solar exposure and not as a measurement of the thermal environment at the pole during the activity season. This limitation is considered in Section 4.1.

Two candidate variables were not entered in the model. Distance to roads was considered in order to control for the co-occurring risk of road mortality, but the available layer contained only national highways and expressways and omitted minor roads. Because distribution lines frequently follow roads, this omission is a limitation rather than a neutral exclusion, and is discussed in Section 4.3. A sensitivity analysis including distance to roads, as computed from the available layer, is reported in Section 3.5. Aspect was not entered as a linear predictor because it is a circular variable, in which 0° and 359° are adjacent in reality but maximally distant numerically.

The northness index, cos(aspect), was not entered in the primary model. Aspect is represented in the model through the solar radiation index, which is computed from slope and aspect together. We did not screen the variable on its association with the outcome, and a sensitivity analysis in which northness is entered alongside the ten variables is reported in Section 3.5.

#### 2.2.3. Descriptive Comparison and Transformation

Before fitting the logistic regression, we examined the distributions of the environmental variables at case poles and at poles with no reported incident. These comparisons are descriptive: no variable was added to or removed from the model on the basis of them, and all ten were entered simultaneously (Section 2.2.4). None of the variables satisfied normality, so we used two-sided Mann–Whitney U tests and computed the absolute rank-biserial correlation, |r| = |1 − 2U/(n_1_n_2_)|, as an effect size. The ten *p*-values were adjusted using the Benjamini–Hochberg procedure to control the false discovery rate. We also examined the skewness of the distance variables. Using the poles with no reported incident as the reference, joint proximity (skewness 4.77), distance to settlements (5.29), and distance to watercourses (3.60) were strongly right-skewed, so a square-root transformation was applied to these three variables under a pre-specified rule of |skewness| > 3. Distance to the nearest pole (skewness −0.02) was left untransformed.

#### 2.2.4. Case–Control Design and Logistic Regression

We applied a case–control design in which poles with a recorded snake electrocution were cases (y = 1) and poles with no reported incident were controls (y = 0) [27,28]. Case poles were identified by matching the coordinates of each incident to the utility pole database and taking the nearest pole. Matching yielded 189 records at 184 poles in Daegu–Gyeongbuk, with 5 poles associated with more than 1 incident. In the training region each incident coordinate was assigned to the nearest pole in the utility pole database. The 148 species-identified incidents analysed in the first submission were matched under a 30 m tolerance. The per-incident matching log from the step that added the remaining incidents was not retained, so we cannot state whether the same tolerance was applied to those records, and we do not assume that it was. In the validation province, where the incident coordinates and the inventory were supplied as separate extracts that do not align exactly, a 50 m threshold was applied and the two incidents with no inventory pole within that distance were left unmatched (Section 3.6). Where more than one pole lay close to an incident coordinate, the nearest was taken, so the assignment of an incident to a particular pole carries uncertainty: the spacing between adjacent poles is of the same order as the offset between the two extracts: the median distance from a pole to its nearest neighbouring pole, which is the predictor POL_DIST and not a matching distance, is 34.6 m at case records and 33.1 m at control poles (Section 3.4). We did not evaluate the direction of any error that misattribution introduces. The incident-to-pole assignment in the training region was made when the environmental variables were first extracted, and the per-incident matching distances from that step are not recoverable from the files retained. The corresponding figures for the validation province, where both extracts were available, are given in Section 3.6.

Of the 449 incidents, 23 carry no coordinate, and all 23 are in Daegu–Gyeongbuk. The spatial analyses in the training region, therefore, use 189 of the 212 incidents recorded there. This is a second form of structured missingness, distinct from variation in species identification and concentrated in the model-training region. We have no information on whether these incidents differ systematically from those with coordinates. Because a pole can be struck more than once, records rather than poles were used as the unit of analysis, and a sensitivity analysis restricted to unique poles is reported in Section 3.5.

Control poles were drawn at random from the full pole inventory, excluding case poles, at 5 times the number of case poles then available and repeated 10 times, giving 7400 control entries. Because the draws were independent, 55 of these entries are repeat draws of a pole already drawn in another replicate. A pole drawn twice would otherwise carry twice the weight of a pole drawn once, which the design does not justify, so we reduced the pooled set to one entry per pole. Of the resulting 7345 distinct control poles, 4 have no slope value and were excluded by the complete-case requirement, leaving the 7341 control poles used in the model. Retaining the repeat draws instead gives 7395 control entries at the same 7341 poles and changes the joint-proximity odds ratio from 0.469 to 0.470, leaving the same 6 variables significant—that variant is reported in Table S9. The per-replicate identifier lists and the random seed of the original draw were not retained, so the draws cannot be reconstructed individually, and the ten 1:5 replicates reported in Table S5 are subsamples of the retained control set rather than fresh draws from the inventory or a reproduction of the original draw. The draw was made before the case set was extended to all records with coordinates, so the realised ratio in the analysis is approximately 39 controls per case record rather than the nominal 5:1 of a single draw. The intercept of a case–control model is determined by the sampling ratio, so the fitted values are relative ranking scores rather than absolute probabilities, and the AUC and the capture rate, which depend only on the ranking, are unaffected by a change in the intercept alone. This is a statement about the intercept of a fitted model and not a claim that a model refitted on a differently constituted control sample would give the same coefficients. The sensitivity of the coefficients to the choice of control poles is examined separately in Tables S5 and S9. No control pole shared coordinates with a case pole. All control poles were used in a single model (Figure 1b).

The logistic regression model was defined as logit(*p*) = β_0_ + Σβ_i_x_i_, where exp(β_i_) is the odds ratio (OR) associated with a one-standard-deviation increase in variable i. All ten variables were standardised to z-scores and entered simultaneously. Confidence intervals and *p*-values were obtained from Wald statistics based on maximum likelihood estimation. Stepwise selection was not used, because of known problems of bias and reproducibility [29]. Multicollinearity was assessed with the variance inflation factor (VIF), with a threshold of 10. The number of events per estimated coefficient, excluding the intercept, was 189/10 = 18.9, which meets conventional guidance [30]. We use this ratio as a guide rather than as a threshold. The influence of the choice of control poles on the coefficients was examined separately using ten 1:5 subsamples of the retained control set (Table S5). This assesses sensitivity to control selection within that set and is not a resampling of the provincial inventory.

Because odds ratios for a square-root-transformed distance are not constant across the range of that distance, we additionally report the change in odds associated with specified increments in the original units (for example from 100 m to 200 m) for the transformed variables, computed from the fitted model (Table S6).

Because the intercept of a case–control model is determined by the sampling ratio rather than by the population prevalence of the event, the fitted values are relative ranking scores for ordering poles and are not absolute probabilities of electrocution. Case–control sampling affects the model intercept but does not invalidate the slope coefficients, and the corresponding odds ratios remain valid measures of relative association. AUC and the capture rate depend on the ranking of the fitted values and are, therefore, unaffected by an intercept-only correction. We report relative association and risk ranking throughout and do not present absolute probabilities of electrocution.

#### 2.2.5. Spatial Dependence and Functional Form

Because electrocution incidents are spatially clustered (Figure 1b), residual spatial dependence could render the model-based standard errors and *p*-values anticonservative. We tested the Pearson residuals of the fitted model for spatial autocorrelation using Moran’s I with row-standardised weights, under 6 weight specifications (the 4, 8, and 16 nearest neighbours, and distance bands of 1, 2, and 5 km), with significance assessed by 999 random permutations. Where residual autocorrelation was present, we recomputed inference using cluster-robust standard errors on a spatial grid, at 5, 10, and 20 km resolutions, and report whether the significance of each variable was preserved. This adjusts the estimated uncertainty of the coefficients for dependence within grid cells—it does not remove spatial structure from the residuals, and it does not address dependence between cells.

We also assessed the assumption that each continuous predictor is linear on the logit scale, by comparing the fitted model with a model in which that predictor was represented by a natural cubic spline with three degrees of freedom, using a likelihood-ratio test. Variables showing departure from linearity were carried into a sensitivity model in which all such variables were entered as splines, and we report whether the conclusions for the remaining variables were preserved. The linear specification was fixed before these diagnostics were run and is retained as the primary model, for interpretability and because the spline model raises the number of estimated coefficients from 10 to 22, so that the number of events per coefficient falls from 18.9 to 8.6. That ratio is a guide rather than a threshold [30], and we do not claim on its basis that the spline coefficients are unreliable. We report the spline model as a sensitivity analysis (Table S8) and state which inferences it preserves and which it does not. We did not compare the two specifications on validation performance, so we do not claim that either is the better predictive model, and the ranking applied to the full inventory uses the linear specification.

#### 2.2.6. Model Validation and Application to the Full Pole Inventory

Model discrimination was evaluated using the area under the receiver operating characteristic curve (AUC) [31]. Internal evaluation was reported in three forms: the apparent AUC obtained on the training data, a fivefold stratified cross-validated AUC, and a spatial block cross-validated AUC in which five blocks were formed by k-means clustering of pole coordinates and each block was withheld in turn, to assess discrimination when spatially grouped observations were withheld. The folds of the fivefold procedure were formed over records rather than poles, so the ten records at the five poles associated with more than one incident can fall on both sides of a split. Repeating the procedure with the folds grouped by pole, so that all records at a pole are withheld together, changes the cross-validated AUC from 0.683 to 0.675. The spatial block procedure, which partitions on coordinates, does not have this property. External validation applied the model fitted in Daegu–Gyeongbuk, including its standardisation parameters, unchanged to Chungbuk. The Chungbuk validation set comprised the 32 case records (at 31 poles) and 260 poles with no reported incident drawn at random from the provincial inventory. This control set was drawn before the model reported here was fitted and was retained unchanged, so the external estimate is not conditioned on the specification finally adopted. Using a set that contains both cases and controls avoids the interpretational problems of evaluating AUC from presence-only data [32]. Ninety-five percent confidence intervals for the apparent and external AUC were obtained by nonparametric bootstrap resampling of the records, not of the poles, with 4000 replicates—replicates in which the resample contained only one class were discarded.

To evaluate the model as a screening tool at an operationally realistic scale, we additionally applied it to the complete Chungbuk pole inventory. All 10 environmental variables were computed at each of the 264,036 poles by the same procedures used for the training data—1389 poles were excluded, leaving 262,647. Every one of the 1389 was excluded for the same reason—the Sentinel-2 composite returned no NDVI value at that location—and no pole was lost for any other variable. We checked whether this exclusion removed poles at which an incident had been recorded, by taking the nearest inventory pole to each incident coordinate from the full inventory before any exclusion, and the result is reported in Section 3.6. Forest cover for the full inventory was computed from a 20 m raster representation of the forest layer using a summed-area table, rather than by polygon intersection, as in the training data. Agreement between the two methods was close: at the 261 of the 292 validation records whose coordinates coincide with an inventory pole, r = 0.999 with a median absolute difference of 0.5 percentage points, and when each of the remaining records is compared with its nearest inventory pole the correlation across all 292 is 0.998 with the same median difference. Poles were ranked by fitted score, and we report the proportion of poles with a recorded incident that fall within the highest-ranked 1%, 5%, 10%, 20%, 30%, and 50%, with exact binomial confidence intervals, benchmarked against the proportion expected under random selection.

We refer to this quantity throughout as the capture rate: the proportion of poles with a recorded incident that are contained within a given highest-ranked subset. It is not an estimate of the proportion of incidents that would be prevented if those poles were retrofitted, because the effectiveness of mitigation was not evaluated in this study.

#### 2.2.7. Timing of Incidents

The time recorded for each incident is the time at which the supply interruption occurred, as distinct from the time at which the fault was notified or attended—this was confirmed with the utility. We, therefore, use it as the timing of the fault-generating contact. It remains a record of when faults occurred rather than an independent observation of animal activity, and incidents that did not generate a recorded fault are absent by construction.

For each incident with a date, a time, and coordinates (425 of 449), we computed sunrise and sunset for that date at that location using the NOAA solar position equations with a solar zenith angle of 90.833° and classified the incident as diurnal or nocturnal accordingly. Because daylight occupies more than half the day during most of the activity season, the observed proportion of diurnal incidents cannot be interpreted without reference to the time available. We, therefore, tested the observed number of daytime incidents against the null hypothesis that each incident occurs during daylight with a probability equal to the proportion of that date and location that was daylight. Because this probability differs between incidents, the null distribution of the total is Poisson-binomial rather than binomial, and we used an exact Poisson-binomial test. The mean daylight proportion across incidents is reported alongside the observed proportion as a descriptive benchmark. For diurnal incidents we additionally expressed the time of the incident as a proportion of the interval between sunrise and sunset at that date and location and tested this against a uniform distribution with a Kolmogorov–Smirnov test.

### 2.3. Statistical Analysis

Spatial data were processed in QGIS 3.40 [33]. Environmental variables were extracted, and the solar radiation index was computed, in Python 3.10 using geopandas, rasterio, shapely, and pvlib. Statistical analyses were performed in Python 3.13 using statsmodels 0.15.0 [34], scikit-learn 1.9.1, SciPy 1.18.1, pandas 3.0.5, and GeoPandas 1.2.0. File S1 contains eleven Python scripts for model fitting and validation, the sensitivity analyses of Tables S5, S6 and S9, the regional, monthly, and diel analyses, spatial-dependence and functional-form diagnostics, the control-set audit, joint-proximity comparisons, full-inventory evaluation, and the three main figures. Input-path and output-directory settings are included at the beginning of each script. The scripts operate on analysis-ready inputs and do not include the restricted incident records, pole inventory, or derived environmental data. Upstream GIS extraction, NDVI compositing, calculation of the solar index, and photographic determinations are outside the scope of File S1. The accompanying README documents software requirements, execution order, path settings, and required input fields, including an input-variable dictionary. No separate configuration file is required.

Descriptive statistics are given as medians with interquartile ranges (IQRs). Two-group comparisons used Mann–Whitney U tests, and *p*-values for multiple comparisons were adjusted using the Benjamini–Hochberg procedure.

Because the extraction window of 1 January 2024 to 30 June 2025 does not contain each calendar month an equal number of times—January to June occur twice and July to December once—the expected frequencies for the monthly test were set proportional to the number of times each month was observed. Monthly frequencies were then tested with a chi-square goodness-of-fit test, restricted to the complete extraction window. Because snakes in this region are inactive for part of the year, a test of uniformity across all twelve months is of limited biological interest. We, therefore, present the test restricted to the activity season (April to October) as the primary result and the twelve-month test in the Supplementary Materials.

Regional frequencies could not be tested against a uniform expectation because the number of poles differs among regions. We, therefore, compared each region with the remainder of the country rather than with a national mean that includes the region itself: for each region we computed the rate per 100,000 poles, the rate ratio relative to all other regions combined, and an exact binomial test of the observed count against the count expected from that region’s share of the national pole inventory, with exact confidence intervals for the rate ratio. The rates are computed from all 447 incidents that fall in the 11 regions for which a pole count was supplied. These incidents span 1 January 2024 to 4 July 2025—the complete 18-month extraction window together with the 3 incidents recorded on 1–4 July 2025—with the exception of 1 record that carries no date and is retained because the comparison requires only the regional headquarters field. We, therefore, describe the period of the rates as the extraction window together with those three incidents rather than as 18 months, and the rates are cumulative recorded incidents per 100,000 poles over that period rather than annualised rates. Restricting the rates to the complete window alone changes only the three regions that contain the July incidents—Chungbuk from 12.12 to 11.74, Gyeongnam from 9.66 to 9.42, and Daejeon–Sejong–Chungnam from 1.34 to 1.15 per 100,000—and leaves the ranking of regions and the Daegu–Gyeongbuk rate ratio essentially unchanged (4.27 against 4.33).

The significance level was α = 0.05, and results are reported as *** *p* < 0.001, ** *p* < 0.01, * *p* < 0.05, and ns (not significant).

## 3. Results

### 3.1. Species Composition and Condition

The 323 incidents for which the species could be identified involved 324 individuals of 6 species (Table 2). Amur ratsnakes (*Elaphe schrenckii*) accounted for 202 individuals (62.3%), steppe ratsnakes (*E. dione*) for 97 (29.9%), and red-banded snakes (*Lycodon rufozonatus*) for 18 (5.6%); together, these three colubrids accounted for 97.8% of the identified individuals. Tiger keelbacks (*Rhabdophis tigrinus*) accounted for five individuals (1.5%), and the two viperid species—rock mamushi (*Gloydius saxatilis*) and Ussuri mamushi (*G. ussuriensis*)—were represented by 1 individual each (0.3%).

Two separate circumstances produced more than one record or individual at a single location, and these should not be confused. In one incident, on 20 May 2025 in Yeongdong, Chungbuk, two steppe ratsnakes were recorded together, and the post-incident photographs showed genital association consistent with mating; therefore, 1 incident corresponds to 2 individuals, which is why the 323 identified incidents comprise 324 individuals. Separately, on 10 July 2024 in Gyeongbuk, two entries were logged at the same pole at the same second, one recording an Amur ratsnake and one a steppe ratsnake, each with its own species determination from a carcass photograph. The register does not carry an identifier for the supply interruption itself, so it cannot be established from it whether these were two interruptions or one interruption involving two animals. We retained both entries as two records, which is the analysis unit throughout, and this is one of the five poles associated with more than one record in the case–control design (Section 2.2.4).

Forty-one individuals showed abdominal features interpreted as possible recent feeding (24 Amur ratsnakes, 16 steppe ratsnakes, and 1 red-banded snake), and 18 showed features interpreted as possible gravidity (9 Amur ratsnakes and 9 steppe ratsnakes), 1 of them with an egg partly extruded. As noted in Section 2.1.2, these are assessments of photographic appearance, made without a scale object and not independently confirmed.

### 3.2. Temporal Patterns

Incidents were recorded throughout the 18-month extraction window, from 9 January 2024 to 30 June 2025, with 3 further incidents on 1–4 July 2025. Four incidents fell between January and March: one on 9 January 2024 in Busan–Ulsan, identified from a photograph as a steppe ratsnake, and three in Gyeongbuk on 19 February, 2 March, and 30 March 2025, none of which carried a usable photograph. Within 2024 the next incident after January was on 28 April. All four were logged in daylight, between 08:56 and 14:18. One species-identified individual carries no recorded date and is, therefore, absent from Figure 2, which accordingly sums to 323 of the 324 identified individuals.

Because January to June were observed twice within the extraction window and July to December once, monthly frequencies were tested against expected counts proportional to the number of times each month was observed. Restricted to the activity season (April to October), frequencies were strongly non-uniform for both the full register (χ^2^ = 490.6, df = 6, *p* < 0.001) and the identified individuals (χ^2^ = 377.7, df = 6, *p* < 0.001). Standardised residuals were strongly positive in June (+16.0 for the register, +14.5 for identified individuals) and positive in July (+6.0 and +3.9) and May (+1.9 and +2.0), and negative in April and from August onwards. In absolute terms May was the second most frequent month (106 incidents in the register, against 238 in June and 84 in July). The corresponding twelve-month tests are given in Table S7; as noted in Section 2.3, these are of limited biological interest for a seasonally inactive taxon and are reported for completeness.

More incidents were recorded in June 2025 (181 in the register) than in June 2024 (57). As set out in Section 2.1.1, we cannot establish whether the coding of outage causes changed between the two years, and this difference should not be read as a measured change in incidence.

#### Timing Within the Day

Of the 449 incidents, 425 had a date, a time, and coordinates and could be classified relative to local sunrise and sunset. Sunrise across these incidents ranged from 05:04 to 07:32 and sunset from 17:28 to 19:59, with day length between 9.9 and 14.8 h.

Among the species-identified incidents, 233 of 322 (72.4%, 95% CI 67.1–77.2) occurred during daylight, against a mean daylight proportion of 60.1% across the dates and locations of those incidents. Under the null hypothesis that each incident occurs during daylight with a probability equal to the daylight proportion for its own date and location, the observed number was higher than expected (exact Poisson-binomial test, one-sided *p* = 2.9 × 10^−6^). The same held for the full register (298 of 425 diurnal, 70.1% observed against a mean of 60.0%, *p* = 8.9 × 10^−6^) and for the activity season alone (69.9% against 60.1%, *p* = 1.8 × 10^−5^).

Within daylight hours, incidents were not spread evenly between sunrise and sunset: expressed as a proportion of that interval, the median incident occurred at 0.44 (IQR 0.32–0.60) and the distribution departed from uniform (Kolmogorov–Smirnov D = 0.194, *p* = 2.4 × 10^−10^). We report this as a description of the register and do not interpret it behaviourally, for the reason given below.

These results describe when outage-generating electrocutions were logged. As noted in Section 2.2.7, the recorded time is the time of supply interruption and not an independently observed time of animal activity, and incidents that generated no recorded fault are absent by construction. The percentages above are, therefore, properties of the register, and we do not convert them into statements about the daily activity of the animals.

### 3.3. Regional Variation in Electrocution Rate

Of the 449 snake-contact incidents, Daegu–Gyeongbuk accounted for the most (212, 47.2%), followed by Gwangju–Jeonnam (52, 11.6%), Northern Gyeonggi (45, 10.0%), Gyeongnam (40, 8.9%), and Chungbuk (32, 7.1%).

After adjusting for the number of poles in each region, and comparing each region with the remainder of the country rather than with a national mean that includes it, the rate was highest in Daegu–Gyeongbuk at 27.84 incidents per 100,000 poles over the 18-month extraction window together with the 3 incidents recorded on 1–4 July 2025, 4.27 times the rate in the rest of the country (95% CI 3.53–5.17, *p* < 0.001). Northern Gyeonggi was also elevated (19.56 per 100,000; rate ratio 2.01, 95% CI 1.45–2.74, *p* < 0.001). Rates were significantly below the rest of the country in Gangwon (5.51; 0.52, 0.31–0.81), Jeonbuk (4.86; 0.45, 0.27–0.71), Gyeonggi (3.62; 0.33, 0.18–0.55), Busan–Ulsan (2.33; 0.22, 0.07–0.52), Daejeon–Sejong–Chungnam (1.34; 0.12, 0.05–0.24), and Incheon, where no incident was recorded. Chungbuk, Gyeongnam, and Gwangju–Jeonnam did not differ significantly from the rest of the country (Table S1). Two incidents recorded in Jeju are excluded from this comparison because a pole count for that region was not supplied.

The ranking of the four highest-rate regions was unchanged when the analysis was restricted to the species-identified incidents, and when incidents with an uncertain cause were excluded (Table S3). The absolute rates, however, are higher in the full register than in the species-identified subset, as they must be, and the increase is not uniform across regions: in Gwangju–Jeonnam, where only 44.2% of incidents could be identified to species, the rate rises from 3.64 per 100,000 in the identified subset to 8.22 in the full register, a factor of 2.26, and the region ceases to be significantly below the rest of the country, whereas in Daegu–Gyeongbuk, with 69.8% identified, it rises from 19.43 to 27.84, a factor of 1.43. Gangwon moves in the opposite direction, from indistinguishable from the rest of the country in the identified subset to significantly below it in the full register. This is a direct consequence of the spatially structured variation in identification rate reported in Section 2.1.1 and is the reason the regional comparison is based on the full register.

### 3.4. Environmental Characteristics of Case and Control Poles

Comparison of the 189 case records (at 184 poles) with the 7341 poles with no reported incident in Daegu–Gyeongbuk showed significant differences in 7 of the 10 variables after FDR adjustment (Table 3; Figure S6). Ranked by effect size, these were joint proximity to forest and farmland (|r| = 0.213), distance to watercourses (0.155), pole density (0.151), NDVI (0.139), elevation (0.130), slope (0.110), and forest cover (0.099). Case poles had forest and farmland closer to them jointly (median 65.7 m, IQR 22.4–134.8) than control poles (103.0 m, 36.8–272.3), and they were also closer to watercourses (522.7 m vs. 753.0 m) and occurred in areas with lower pole density (71.0 vs. 78.0 km^−2^), higher forest cover (24.8% vs. 14.6%), higher NDVI (0.5 vs. 0.4), higher elevation (115.0 m vs. 92.0 m), and steeper ground (5.2° vs. 4.3°).

Distance to settlements and distance to the nearest pole did not differ significantly in this unadjusted comparison (*p* = 0.445 and *p* = 0.232), although both were significant after adjustment for the other variables (Section 3.5). Annual solar radiation did not differ in either the unadjusted or the adjusted comparison.

### 3.5. Logistic Regression

Six of the ten variables were significant in the multivariable model (Figure 3; Table S4). The odds of electrocution decreased with pole density (OR = 0.40, 95% CI 0.26–0.61), joint proximity to forest and farmland (0.47, 0.32–0.69), distance to settlements (0.55, 0.43–0.71), forest cover (0.68, 0.53–0.87), and distance to watercourses (0.80, 0.68–0.94), and increased with distance to the nearest pole (1.16, 1.04–1.28). Elevation, annual solar radiation, slope, and NDVI were not significant. The maximum VIF was 2.62 and the number of events per variable was 18.9.

Because odds ratios for square-root-transformed distances are not constant across the range of the variable, effects in the original units are given in Table S6. For joint proximity, an increase in FF_PROX from 0 to 100 m corresponded to an odds ratio of 0.51, and a further increase from 100 m to 200 m to an odds ratio of 0.76.

Distance to settlements and distance to the nearest neighbouring pole were not significant in the unadjusted comparisons but were significant in the multivariable model. Forest cover was significant in both comparisons, but the associations had opposite directions: case poles had higher surrounding forest cover than control poles (median 24.8% versus 14.6%; unadjusted *p* = 0.027), whereas higher forest cover was associated with lower odds in the fitted model (OR = 0.68; adjusted *p* = 0.002). Forest cover and joint proximity were correlated on the transformed scale (r = −0.47). These differences between unadjusted and adjusted associations are considered in Section 4.2.

#### 3.5.1. Spatial Dependence and Functional Form

The Pearson residuals of the fitted model showed significant positive spatial autocorrelation under all six weight specifications tested (Moran’s I between +0.044 and +0.090; permutation *p* = 0.001 in each case). Recomputing inference with standard errors clustered on a spatial grid preserved the significance classifications: the same six variables were significant at 5 km, 10 km, and 20 km resolutions as under model-based standard errors, although forest cover and distance to watercourses weakened from *p* < 0.01 to *p* < 0.05 at the 20 km resolution (Table S4).

Six continuous predictors departed from linearity on the logit scale, tested by comparing each in turn with a natural cubic spline of three degrees of freedom: distance to the nearest pole (likelihood-ratio χ^2^ = 23.9, df = 2, *p* < 0.001), elevation (22.1, *p* < 0.001), pole density (10.8, *p* = 0.004), slope (9.8, *p* = 0.007), distance to settlements (9.7, *p* = 0.008), and distance to watercourses (6.4, *p* = 0.041). The remaining four, including joint proximity (1.9, *p* = 0.40), did not. In a sensitivity model entering all six non-linear predictors as splines, joint proximity, pole density, distance to settlements, distance to watercourses, and distance to the nearest pole remained significant, and elevation became significant through its non-linear terms. Forest cover lost significance (*p* = 0.068), and slope and NDVI remained non-significant, as in the linear model. The spline specification improved in-sample fit (AIC 1616.8 against 1671.4) and reduced, but did not eliminate, residual spatial autocorrelation (Moran’s I decreased from +0.051 to +0.035 under the eight-nearest-neighbour specification). The linear model was retained as the primary specification for interpretability, with the spline results reported in full as a sensitivity analysis (Table S8). Validation performance was not compared between these specifications.

#### 3.5.2. Sensitivity Analyses

The results were stable across the analysis sets and model variants defined in Section 2.1.1 (Table S9). The odds ratio for joint proximity ranged from 0.40 to 0.49 and was significant at *p* < 0.001 in every variant: taking 1 record per case pole rather than 1 row per record (0.47), excluding the 7 incidents with an uncertain cause (0.45), excluding all 9 (0.46), restricting to the 148 species-identified incidents (0.41), retaining the northness index (0.47), adding distance to roads from the incomplete road layer (0.40), adding an edge-density index derived from NDVI thresholds (0.49), and retaining the repeat draws in the pooled control set (0.47). The number of significant variables was six of ten in all but two variants: seven in the species-identified subset, in which elevation also reached significance, and five when the NDVI-derived index was added. Across ten 1:5 subsamples of the retained control set, the sign of every coefficient that was significant in the full model agreed with the full model in all ten replicates (Table S5).

### 3.6. Model Validation and Spatial Transferability

The apparent AUC was 0.701 (95% CI 0.671–0.731), the fivefold cross-validated AUC 0.683, and the spatial block cross-validated AUC 0.654 (Table 4; Figure S7). Applying the model fitted in Daegu–Gyeongbuk unchanged to the Chungbuk validation set (32 case records at 31 poles, and 260 poles with no reported incident) gave an AUC of 0.683 (95% CI 0.586–0.771).

The model was then applied to the complete Chungbuk pole inventory. Of the 32 incidents recorded in the province, 30 lay within 50 m of an inventory pole and resolved to 29 distinct poles, and the remaining 2 had no inventory pole within 50 m and could not be matched. Across the 262,647 of the 264,036 inventory poles at which all 10 variables could be computed (the 1389 excluded are those for which the Sentinel-2 composite returned no NDVI value), the AUC was 0.620 (bootstrap 95% CI 0.531–0.704, obtained by resampling the 29 matched incident poles with replacement 4000 times). None of the 32 incidents in the province was lost through the exclusion of poles with missing NDVI. Taking the nearest inventory pole to each incident coordinate from the full inventory of 264,036 poles before any exclusion, the 32 incidents resolve to 31 distinct poles, and every one of those 31 carries an NDVI value and a complete set of the other 9 variables. This holds both for the 29 poles to which the 30 incidents within 50 m resolve and for the nearest poles to the 2 incidents that could not be matched, which lie 51.4 m and 58.7 m away. The two unmatched incidents are, therefore, excluded by the 50 m distance criterion and not by missing data, and the two exclusion routes do not overlap. The capture rate—the proportion of poles with a recorded incident contained within a given highest-ranked subset—was 3.4% within the highest-ranked 1% of poles (95% CI 0.1–17.8), 3.4% within the highest-ranked 5% (0.1–17.8), 13.8% within 10% (3.9–31.7), 27.6% within 20% (12.7–47.2), 44.8% within 30% (26.4–64.3), and 65.5% within 50% (45.7–82.1), against 1%, 5%, 10%, 20%, 30%, and 50% expected under random selection. The point estimates exceed the random-selection benchmark at every threshold except the highest-ranked 5%, where a single incident pole was captured against the 1.45 expected by chance—the highest-ranked 1% and 5% subsets contained the same single pole. The confidence intervals include the random-selection benchmark at every threshold, so these estimates do not establish that the ranking outperforms random selection across the inventory.

A similar unadjusted difference in joint proximity was observed in this province: the 29 poles matched to an incident had a median joint-proximity value of 57.8 m, against 90.7 m for the remaining 262,618 poles (Mann–Whitney *p* = 0.010, |r| = 0.275).

All of these capture rates carry wide confidence intervals, because only 29 poles in the province were matched to a recorded incident. They are also affected by uncertainty in matching: incident coordinates and the pole inventory are supplied as separate extracts and do not align exactly, with a median separation of 31.9 m between an incident coordinate and the nearest inventory pole (90th percentile 48.3 m, maximum 58.7 m), against a median spacing of 37.3 m between adjacent poles. Here, 5 of the 32 incident coordinates have a second inventory pole within 30 m, so some incidents may have been attributed to a neighbouring pole. A misattributed pole may rank above or below the correct one, and we did not evaluate the direction or the magnitude of this effect.

As set out in Section 2.2.6, the capture rate describes the ability of the model to rank poles at which incidents were recorded and is not an estimate of the proportion of incidents that retrofitting those poles would prevent.

## 4. Discussion

### 4.1. Which Snakes Are Electrocuted, and Why

Three colubrid species accounted for 97.8% of the individuals recorded, whereas viperids were rarely represented. This pattern is consistent with differences in movement behaviour, climbing ability, and body form among these taxa [20], although we have no independent estimate of the abundance or of the exposure of each species in the study region, and therefore cannot convert the observed composition into a species-specific risk.

Morphology and locomotor ability point in the same direction. Snakes ascend vertical structures by concertina or arboreal locomotion [21,22], both of which require a body long enough to span successive purchase points and musculature able to support a cantilevered anterior body, and studies of snake road mortality indicate that the more mobile species are the more likely to encounter linear infrastructure [16,17]. *Elaphe schrenckii*, *E. dione*, and *Lycodon rufozonatus* are all capable climbers that are regularly recorded above ground, whereas the two *Gloydius* species are short-bodied terrestrial ambush predators. Body length also governs whether an animal that reaches energised equipment can complete a circuit: an individual must bridge two conductors, or a conductor and an earthed component, for a fault to occur, which is how climbing behaviour and access to power infrastructure translate into electrical faults [8]. The three colubrids differ considerably in size. *L. rufozonatus* is a medium-sized species, and its presence in the sample indicates that the capacity to bridge pole-mounted equipment is not confined to the largest snakes. Tiger keelbacks (*Rhabdophis tigrinus*, five individuals) are likewise neither large nor strongly arboreal, and their appearance in the register suggests that occasional climbing by less specialised species also results in electrocution.

The immediate motivation for climbing cannot be determined from our data, which consist of post hoc records of animals already dead. Birds nesting or perching on pole-mounted equipment are a plausible attractant, since the supporting structures of power infrastructure are used as nest sites by birds [35], as are rodents sheltering in the structure. Thermoregulation on sun-exposed metal is a further possibility, because snakes exploit the thermal environment of artificial structures [18], and the steppe ratsnake in particular has been shown to select artificial structures as refuges and thermoregulation sites [19]. We did not measure any of these, and the solar radiation index used here is a coarse terrain-derived quantity rather than a measurement of the thermal environment at the pole during the activity season (Section 2.2.2). Its lack of association with electrocution should not be read as evidence against a thermal mechanism, which this dataset cannot test.

Age composition could not be assessed reliably from these photographs. The photographs contain no scale object, so body length could only be estimated against infrastructure in the same frame, and no defensible species-specific length threshold for maturity can be applied to such an estimate (Section 2.1.2). Had an age composition been available, two mechanisms would in any case have been difficult to separate: juveniles may climb less or may be less able to span two conductors and so less likely to generate the fault that brings an incident into the register at all, which would make the age composition a property of detection rather than of mortality. The features interpreted as possible recent feeding or possible gravidity, and the genital association in the one pair, rest on criteria that can be stated and illustrated (Section 2.1.2, Figure S5), but they are observations of photographic appearance rather than verified physiological states, and we did not measure any population-level effect.

Four incidents fell within the period in which snakes in this region are normally inactive, one in January and three in February and March, and they deserve a brief comment because they are not expected under a simple account of surface activity. All four were logged in daylight, and the January record is from Busan–Ulsan on the warm southern coast. Two explanations are consistent with what the register contains. A snake may have been active at the surface on an unseasonably warm day, which is plausible on the southern coast in January but less so in inland Gyeongbuk in February; alternatively, an animal overwintering within the pole-mounted equipment itself may have come into contact with an energised part, since the enclosures and cavities of distribution equipment offer insulated refuges above the frost line. The register records neither the behaviour preceding contact nor the ambient temperature at the pole, so we cannot distinguish the two, and only one of the four carries a photograph. We note the records rather than interpret them and point out that establishing whether snakes overwinter in pole-mounted equipment would require inspection of the equipment itself rather than analysis of outage records.

### 4.2. Landscape Context of Electrocution

The significant variables collectively describe a landscape setting—their associations should not be interpreted as independent causal effects. The clearest single result is that electrocution was associated with joint proximity to forest and farmland: an increase in FF_PROX from 0 to 100 m was associated with an odds ratio of about 0.5, holding the other variables constant, and the association persisted across the sensitivity specifications we examined and under the cluster-robust and spline specifications. A similar unadjusted difference in the same index was observed in Chungbuk, where poles with a recorded incident had a lower median value than the remainder of the provincial inventory. This is a comparison of the raw distribution and not an independent re-estimation of the adjusted effect.

The value of this index is that it carries the association in both the unadjusted and the adjusted comparison, and in both study regions, which an inference from the adjusted sign of forest cover alone could not do, because the unadjusted and adjusted relationships of forest cover run in opposite directions. We emphasise, however, that the index measures joint proximity to the two land-cover classes and not the distance to a boundary they share (Section 2.2.2). The association is consistent with a forest–farmland edge mechanism but does not test one directly, and measuring the shared boundary itself remains the step that would.

Forest cover retains a reversed sign between the unadjusted and adjusted comparisons, and the reason for this difference remains unresolved. Once joint proximity is held constant, additional surrounding forest is associated with lower odds, which is consistent with poles embedded in continuous forest being less exposed than poles in a forest–farmland mosaic, but forest cover and joint proximity are correlated in this landscape, and the adjusted coefficient should be read as conditional on joint proximity rather than as an independent effect of forest. It is also the one significant habitat variable that does not survive the spline specification (Section 3.5).

Two network variables point to the same conclusion from the engineering side. The odds of electrocution were higher where the pole network was sparse—lower pole density and greater distance to the nearest neighbouring pole—which is consistent with incidents occurring in more sparsely distributed parts of the network. We did not analyse network topology or the position of poles within feeder branches, so we cannot say that these are the outer branches of the distribution system. Pole density differed between case and control poles before adjustment, but distance to the nearest pole did not (Section 3.4), so the adjusted association should be interpreted as conditional on the other variables. Taken together, these results associate recorded incidents with a sparse network where forest and farmland are nearby and settlements are within reach—they do not establish a separate causal effect for each variable.

The direction of the settlement association deserves comment, because it is not what a simple remoteness account would predict. Conditional on the other variables, the odds of electrocution fell as distance to settlement increased: the odds at a pole 100 m from the nearest built-up area were 0.59 times those at a pole on its edge (Table S6). Settlement distance did not differ between case and control poles before adjustment, so this is a conditional association in the multivariable model—it is correlated with pole density, although we did not test which variable produces the change. Within parts of the network of comparable density, poles nearer to settlement were more often associated with an incident. Two readings are compatible with these data and we cannot separate them: the farmland–forest setting represented by the joint-proximity index lies close to villages in this landscape, so settlement proximity may be measuring the same setting from a different direction, and faults on lines serving inhabited areas are more likely to be noticed, reported, and attended, so part of the association may belong to detection rather than to occurrence. The second reading is a specific instance of the general limitation set out in Section 4.3.

Proximity to watercourses showed a weaker association in the same direction. Riparian corridors concentrate amphibian and small-mammal prey and provide movement corridors for the Amur ratsnake [11], so the association is interpretable, but the effect size is small, and we do not build on it.

### 4.3. Reporting, Predictive Performance, and Limitations

Because incidents enter the record only when contact generates a fault, the dataset describes outage-confirmed electrocution and not the full extent of snake mortality on power infrastructure. A fatal contact that produces no recorded fault does not appear in the register. The quantities we report are, therefore, properties of the register, rather than estimates of total snake mortality.

Four features of the register qualify the figures further.

First, the species could be identified for only 323 of the 449 incidents, and the proportion identified varied among the 10 regions with at least 5 incidents from 44.2% to 100%, more than would be expected by chance. Restricting the spatial and regional analyses to species-identified incidents would, therefore, have introduced spatially structured missingness, and the consequences are visible: the regional rate for Gwangju–Jeonnam, where identification was least complete, is more than twice as high in the full register as in the identified subset, and the region changes from significantly below the rest of the country to indistinguishable from it. The regional and spatial analyses are accordingly based on the full register, with the identified-only analysis reported as a sensitivity analysis.

Second, nine records carry field notes indicating a different or uncertain cause—four bird contact, two tree contact, one presumed snake contact, one suspected roadkill, and one that appears to concern substation equipment rather than a pole. Excluding them as the two groups defined in Section 2.1.1—the seven with an uncertain or different cause in the field notes, and those seven together with the further two identified from the remarks field—changed no conclusion (Section 3.5). We did not exclude them one at a time. Their presence indicates that the cause field is not error-free.

Third, we cannot establish whether the procedures for coding an outage cause as snake contact remained unchanged across the study period. The register contains considerably more June incidents in 2025 than in 2024, and we are not able to distinguish a change in incidence from a change in reporting or classification practice. We have, therefore, refrained from interpreting the between-year difference.

Fourth, the seasonal concentration is similar in the full register and in the species-identified subset, and the proportion identified to species was similar between years in the main season, which suggests that variation in photograph availability alone is unlikely to explain it. We emphasise, however, that this does not exclude an influence of photograph availability. It does not establish that the pattern reflects seasonal variation in mortality rather than in recording. Four distinct quantities need to be kept apart: seasonal variation in photograph availability, in the recording of incidents by the utility, in outage-confirmed electrocution, and in snake mortality on power infrastructure overall. Our data speak to the first three and not to the fourth.

The exclusion of distance to roads is a limitation rather than a neutral choice. Distribution lines frequently follow roads, so a road variable would carry information about network placement as well as about road mortality—the available layer omitted minor roads and could not represent this. Including the incomplete layer did not change the conclusions (Section 3.5), but the question cannot be settled with the data available.

The recorded time of an incident is the time at which the supply interruption occurred, confirmed with the utility as distinct from the notification or attendance time (Section 2.2.7), and for a fault-generating electrocution it is used here as the timing of the fault-generating contact. It is still not an independent observation of animal activity: incidents producing no recorded fault are absent by construction, so the register samples the subset of contacts severe enough to trip the line. With that qualification, incidents occurred during daylight more often than the length of the day would predict, so the daytime concentration is not simply a consequence of daylight occupying more than half of the day. We report the distribution within daylight as a property of outage-generating contacts and do not extend it to the activity of the populations from which they are drawn.

Predictive performance was modest and, in our view, is the principal constraint on the practical use of the model. The fivefold cross-validated AUC was 0.683 and the spatial block cross-validated AUC 0.654, and the external AUC in Chungbuk was 0.683 (95% CI 0.586–0.771). Applied to the complete Chungbuk pole inventory, discrimination was lower still (AUC 0.620) and the highest-ranked 10% of poles contained 13.8% of the poles at which an incident had been recorded, against 10% expected by chance. The confidence intervals around these capture rates are wide, because only 29 poles in the province could be matched to a recorded incident, and the matching is itself imprecise: incident coordinates and the pole inventory are separate extracts whose median separation (31.9 m) is comparable to the spacing between adjacent poles (37.3 m), so some incidents may have been attributed to a neighbouring pole. A misattributed pole may rank above or below the correct one, so we cannot say in which direction this affects the estimated capture rates. The gain over random selection across a full inventory is in any case small relative to its uncertainty.

Residual spatial autocorrelation was present in the fitted model. The principal associations remained statistically supported under the cluster-robust covariance specifications examined, but residual spatial dependence persisted, and these sensitivity analyses do not rule out bias from unmeasured, spatially structured factors. The reduction in Moran’s I under the spline specification suggests that functional form may contribute to the residual pattern, although we have not separated that contribution from spatial structure. The *p*-values of the linear model should be read as approximate rather than exact.

Several further limitations apply. We could not include prey distribution, microclimate at the pole, or equipment attributes, such as crossarm or insulator type, because the register records the pole identifier, address, and coordinates but no equipment information, although such characteristics can directly determine electrocution risk [3,4]. Equipment configuration is likely to matter and is a priority for future work with the utility. The incident register covers one country over an 18-month extraction window, with three additional early-July records, and spatial model development and validation focus on two provinces. Our evaluation provides limited evidence of spatial transferability between the two inland provinces examined, and performance in other landscape settings remains uncertain, since the performance of models of this kind often deteriorates markedly when they are transferred to geographically independent areas [36,37,38].

### 4.4. Conservation and Management Implications

For the outage-confirmed electrocution events represented in this dataset, wildlife mortality and supply interruption occur at the same poles. This alignment is the basis of the study’s management relevance: mitigation that proved effective against snake contact could benefit both wildlife conservation and supply reliability at the same pole. We did not evaluate the effectiveness, the magnitude, or the cost-effectiveness of any such measure.

The criterion this study can supply is model-based prioritisation, and its strength should be stated carefully. Across the complete pole inventory of an independent province, the highest-ranked 30% of poles contained 44.8% of the poles at which an incident had been recorded, and the highest-ranked 10% contained 13.8%. These point estimates exceed the proportions expected under random selection, but the uncertainty is substantial and the confidence intervals include those benchmarks at every threshold. We, therefore, describe the model as a possible aid to exploratory prioritisation for field assessment, not as a basis for allocating retrofitting directly, and its operational value would need to be established by further evaluation. A plausible use is to narrow a provincial inventory of a quarter of a million poles to a subset within which inspection and local knowledge can be applied, rather than to identify the specific poles to treat. Used in that way, the ranking is a starting point for field assessment rather than a substitute for it, and the assessment is what would refine it: field inspection of a ranked subset would record the equipment configuration, the climbing route, and the immediate surroundings of each pole, none of which the register contains, and would return the one quantity the present design cannot supply—what is at a high-ranked pole where no incident has been recorded. Future collaboration with the utility could evaluate the ranking through targeted field inspection and prospective monitoring. We regard the model presented here as a first iteration to be revised against data of that kind rather than as a finished instrument.

Two further cautions follow from the design. The capture rate describes the ability of the model to rank poles at which incidents were recorded. It is not an estimate of the proportion of incidents that retrofitting those poles would prevent, because the effectiveness of mitigation against climbing snakes was not evaluated here. The landscape associations describe where recorded incidents occurred over 18 months, not where they will occur next, and a pole with no recorded incident is not thereby a safe pole.

Mitigation measures developed for birds—insulating covers on energised components, anti-perching devices, and increased phase separation—are established in the avian context [2,3], but may or may not be effective against a snake ascending the pole itself. Devices that address the approach to the equipment, such as smooth pole guards, address the climbing route rather than the perching surface and may be more directly relevant. Whether any of these reduce snake electrocution in practice is untested in this system and requires a controlled field trial before investment at scale.

From a conservation perspective, the finding that 62.3% of identified individuals were Amur ratsnakes, a nationally designated endangered species, is notable because it indicates that distribution infrastructure intersects the habitat of a protected species across a wide area. We note, however, that without data on the abundance and exposure of each species we cannot say whether this species is disproportionately at risk or simply the most frequently encountered large colubrid in the regions where electrocution is recorded. Snake life histories are sensitive to increases in adult mortality because maturity is late and annual reproductive output is limited [12,13]. The 18 individuals showing features interpreted as possible gravidity, and the single pair with genital association, suggest that some of the mortality may fall on reproductively active individuals. These are photographic assessments that were not independently confirmed (Section 4.1), and we did not measure any population-level effect.

## 5. Conclusions

Outage-confirmed snake electrocution on utility poles in the Republic of Korea showed marked variation in species composition, season, and region. Three colubrid species accounted for 97.8% of the 324 individuals identified, and 62.3% were Amur ratsnakes, a nationally endangered species. Almost all incidents fell between April and October, with a pronounced June peak. Four incidents fell between January and March, of which one, in January, was identified to species. The same seasonal concentration was present in the complete register of 449 incidents and in the species-identified subset, which suggests that it is not produced by photograph availability alone, although it cannot be separated from seasonal variation in reporting. After adjusting for the number of poles, the rate in Daegu–Gyeongbuk was 4.27 times that in the rest of the country (95% CI 3.53–5.17).

In the case–control model for Daegu–Gyeongbuk, the odds of electrocution were higher at poles with forest and farmland both close by, in sparse parts of the distribution network, near settlements, and near watercourses. The association with joint proximity persisted across the sensitivity specifications examined, including the exclusion of uncertain records, the choice of analysis unit, cluster-robust standard errors, and a more flexible functional form. A similar unadjusted difference was observed in an independent province, although this does not constitute an independent replication of the adjusted effect. The fivefold cross-validated AUC was 0.683, the spatial block cross-validated AUC 0.654, and the AUC in the independent province of Chungbuk 0.683 (95% CI 0.586–0.771).

Applied to the complete pole inventory of that province, the highest-ranked 30% of poles contained 44.8% of the poles at which an incident had been recorded, against 30% expected under random selection. The confidence interval for this proportion (26.4–64.3%) includes the random-selection benchmark, as it does at the other thresholds. Model-based prioritisation may, therefore, assist in exploratory selection of poles for field assessment rather than selection for treatment, and its operational value requires further evaluation. Refining it will require what the incident register does not hold—the equipment configuration at each pole, the climbing route, and observations at high-ranked poles where no incident has been recorded—and collaboration with the utility would be the way to obtain it.

The two objectives are aligned: the poles at which an endangered snake is electrocuted are the poles at which supply is interrupted, so a single intervention could serve both wildlife conservation and network reliability—provided that mitigation measures are first shown to be effective against climbing snakes, which this study did not test.

## Figures and Tables

**Figure 1:**
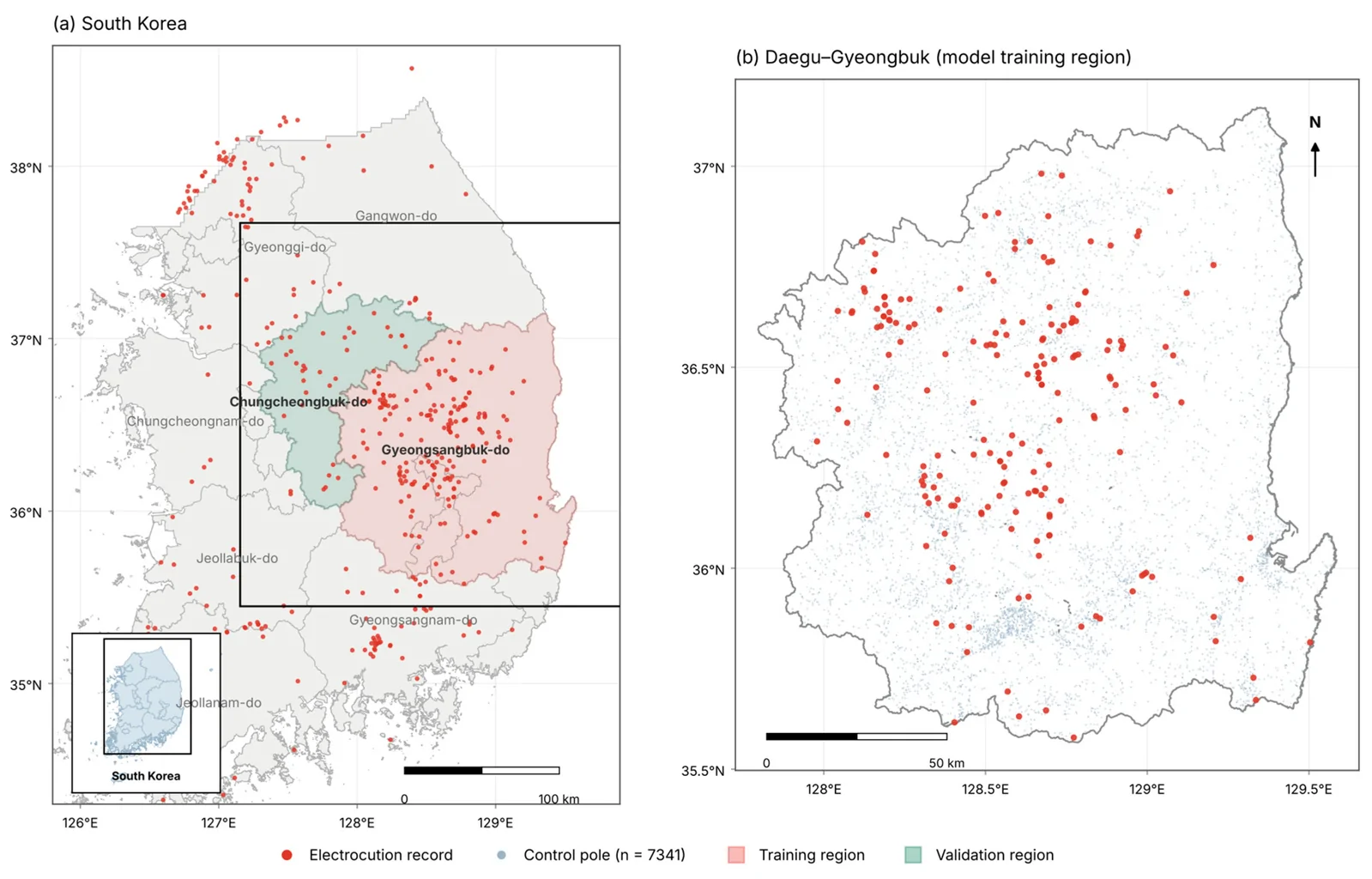
(**a**) Distribution of the 426 snake electrocution incidents with coordinates recorded on utility poles in the Republic of Korea, January 2024–July 2025, with the model training region (Daegu–Gyeongbuk) and the external validation region (Chungcheongbuk-do) shaded. The black rectangle marks the extent of panel (**b**). (**b**) Case–control design in Daegu–Gyeongbuk: the 189 electrocution records (at 184 poles) and the 7341 control poles drawn from the provincial pole inventory. Provinces are labelled in English. Jeju-do and the far-offshore islets of Ulleungdo and Dokdo lie outside the map frames, and two incidents recorded in Jeju are, therefore, not shown, and are excluded from the regional rate comparison because a pole count was not supplied for that region (Section 3.3). Administrative boundaries are from the continuous digital topographic map of the National Geographic Information Institute. Graticule in WGS84, and coordinates projected to EPSG:5186.

**Figure 2:**
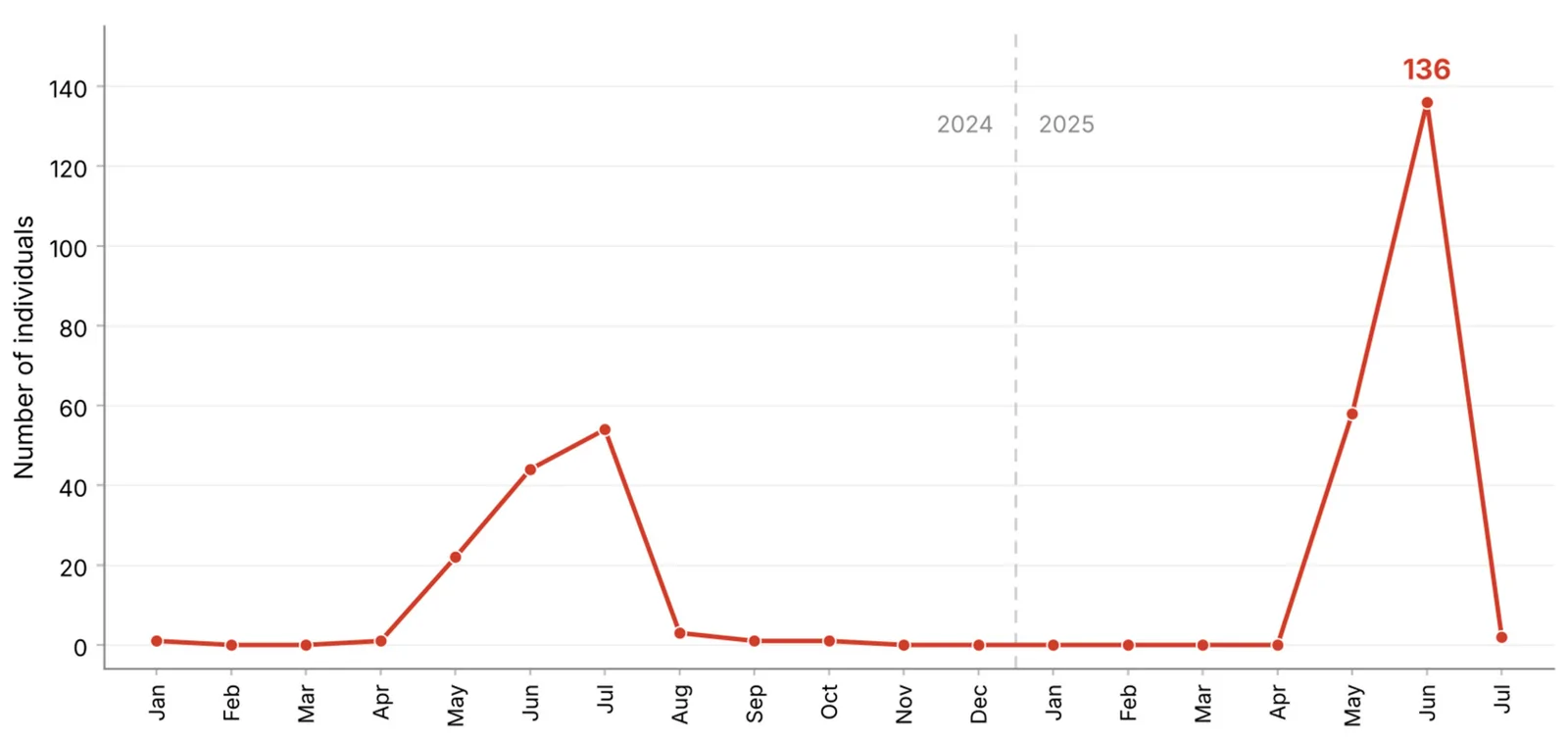
Monthly number of species-identified snakes electrocuted on utility poles in the Republic of Korea (all six species), January 2024–July 2025. The incident register covers this full period. The earliest species-identified incident was recorded on 9 January 2024 and the latest on 4 July 2025, and the next after January was on 28 April 2024. One identified individual carries no recorded date and is, therefore, absent, so the figure sums to 323 of the 324 identified individuals (Table S7). July 2025 contributes records from 1–4 July only and is, therefore, a partial month, so its count is not directly comparable with those of the complete months. The monthly frequency tests in Table S7 are restricted to the complete extraction window and exclude these records.

**Figure 3:**
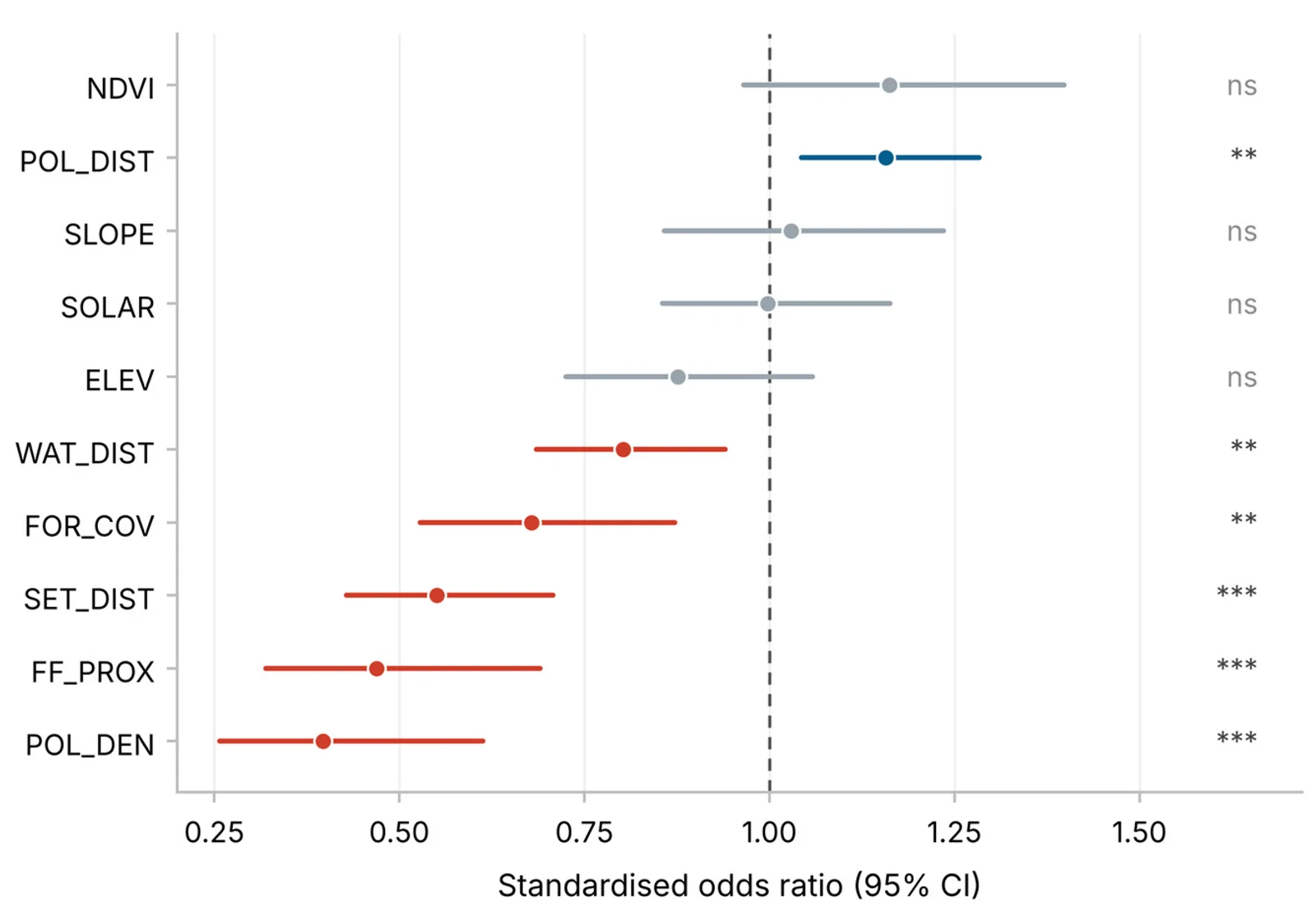
Standardised odds ratios and 95% confidence intervals for electrocution occurrence in the multivariable logistic regression model (189 case records at 184 poles vs. 7341 control poles). All variables were standardised to z-scores, so odds ratios refer to a one-standard-deviation increase. FF_PROX, SET_DIST, and WAT_DIST were square-root-transformed before standardisation. FF_PROX is a joint-proximity index and not a measurement of a mapped forest–farmland boundary (Section 2.2.2). Bars are Wald confidence intervals, and the significance marks are the corresponding model-based *p*-values. The cluster-robust *p*-values are given in Table S4. Red, OR < 1 and significant; blue, OR > 1 and significant; grey, not significant. *** *p* < 0.001; ** *p* < 0.01; ns, not significant; OR, odds ratio; CI, confidence interval. Coefficients, standard errors, variance inflation factors, and spatially clustered *p*-values are given in Table S4, effects in the original units are given in Table S6, and variable codes are defined in Table 1.

**Table 1:** Environmental variables entered in the logistic regression model (*n* = 10).

Code	Variable	Unit	Source	Definition
FF_PROX	Joint proximity to forest and farmland	m	Land-cover map (L2)	Larger of the distances to the nearest forest and the nearest farmland polygon; the radius of the smallest circle centred on the pole containing both classes. A proxy for the setting in which forest and farmland adjoin, not the distance to a boundary they share (Section 2.2.2)
POL_DEN	Pole density	km^−2^	KEPCO pole database	Number of poles in a 1 km × 1 km window centred on the pole
SET_DIST	Distance to settlement	m	Land-cover map (L2)	Euclidean distance to the nearest built-up polygon (classes 110–160)
FOR_COV	Forest cover	%	Land-cover map (L2)	Proportion of forest within a 200 m radius
WAT_DIST	Distance to watercourse	m	National stream network	Euclidean distance to the nearest watercourse
ELEV	Elevation	m	30 m DEM	Elevation at the pole
SOLAR	Annual solar radiation	kWh m^−2^ yr^−1^	30 m DEM + pvlib	Plane-of-array irradiance on a surface with the terrain tilt and azimuth, summed over twelve monthly clear-sky values
SLOPE	Slope	°	30 m DEM	Terrain slope (Horn)
POL_DIST	Distance to nearest pole	m	KEPCO pole database	Euclidean distance to the nearest other pole
NDVI	Vegetation index	–	Sentinel-2 (2024)	(NIR − Red)/(NIR + Red)

Forest comprises land-cover classes 310, 320, and 330, farmland 210–250, and settlement 110–160. FF_PROX replaces the separate distances to forest and to farmland, with which it is collinear (r = 0.92 and 0.83; entering all three together raises the maximum VIF to 18.2). Excluded: distance to roads (minor roads missing from the available layer; sensitivity analysis in Section 3.5), aspect (circular variable), and the northness index cos(aspect) (sensitivity analysis in Section 3.5). DEM, digital elevation model; KEPCO, Korea Electric Power Corporation.

**Table 2:** Species composition of snakes electrocuted on utility poles (*n* = 324 individuals).

Common Name	Scientific Name	Individuals	%
Amur ratsnake	*Elaphe schrenckii*	202	62.3
Steppe ratsnake	*Elaphe dione*	97	29.9
Red-banded snake	*Lycodon rufozonatus*	18	5.6
Tiger keelback	*Rhabdophis tigrinus*	5	1.5
Rock mamushi	*Gloydius saxatilis*	1	0.3
Ussuri mamushi	*Gloydius ussuriensis*	1	0.3
Total		324	100.0

Of the 323 species-identified incidents, 1 involved 2 steppe ratsnakes, so the number of individuals exceeds the number of incidents by 1. Scientific names follow the National Species List of Korea.

**Table 3:** Comparison of environmental variables between case records (*n* = 189, at 184 poles) and poles with no reported incident (*n* = 7341) in Daegu–Gyeongbuk.

Variable	Unit	Case Records, Median (IQR)	Control Poles, Median (IQR)	|r|	*p*	Sig.
FF_PROX	m	65.7 (22.4–134.8)	103.0 (36.8–272.3)	0.213	<0.001	***
WAT_DIST	m	522.7 (193.3–1134.5)	753.0 (271.8–1553.2)	0.155	0.001	**
POL_DEN	km^−2^	71.0 (49.0–91.0)	78.0 (50.0–135.0)	0.151	0.001	**
NDVI	–	0.47 (0.35–0.60)	0.42 (0.26–0.58)	0.139	0.003	**
ELEV	m	115.0 (80.0–169.0)	92.0 (53.0–179.0)	0.130	0.004	**
SLOPE	°	5.2 (3.0–9.2)	4.3 (2.2–9.0)	0.110	0.016	*
FOR_COV	%	24.8 (5.4–37.2)	14.6 (0.0–40.5)	0.099	0.027	*
POL_DIST	m	34.6 (23.8–43.1)	33.1 (23.1–41.7)	0.056	0.232	ns
SET_DIST	m	23.5 (3.4–93.5)	32.4 (0.1–209.2)	0.035	0.445	ns
SOLAR	kWh m^−2^	2249.6 (2188–2349)	2264.6 (2201–2326)	0.020	0.642	ns

Mann–Whitney U tests. *p*-values are Benjamini–Hochberg FDR-adjusted. |r|, absolute rank-biserial correlation; FDR, false discovery rate; IQR, interquartile range; Sig., significance level. Medians and IQRs are given in the original units, before the square-root transformation applied to FF_PROX, SET_DIST, and WAT_DIST. SET_DIST and POL_DIST were not significant in this unadjusted comparison but were significant after adjustment (Section 3.5). FOR_COV is significant here in the opposite direction to its adjusted coefficient. *** *p* < 0.001; ** *p* < 0.01; * *p* < 0.05; ns, not significant. Variable codes are defined in Table 1.

**Table 4:** Discrimination performance of the electrocution risk model.

Validation	Region	AUC	95% CI	Case Units (*n*)	Control Poles (*n*)
Apparent (training data)	Daegu–Gyeongbuk	0.701	0.671–0.731	189	7341
Fivefold cross-validation	Daegu–Gyeongbuk	0.683	–	189	7341
Spatial block cross-validation	Daegu–Gyeongbuk	0.654	–	189	7341
External validation	Chungbuk	0.683	0.586–0.771	32	260
Full pole inventory	Chungbuk	0.620	0.531–0.704	29	262,618

Case units are incident records in the training and external case–control evaluations, but distinct matched incident poles in the full-inventory evaluation. In the full-inventory analysis, 29 incident poles and 262,618 non-incident poles comprise the 262,647 poles with complete covariate data. Full-inventory AUC 0.620 (bootstrap 95% CI 0.531–0.704, resampling the 29 matched incident poles). Capture rate within the complete Chungbuk pole inventory (262,647 poles with all variables computable, of which 29 were matched to a recorded incident): highest-ranked 1% → 3.4% of incident poles (95% CI 0.1–17.8), 5% → 3.4% (0.1–17.8), 10% → 13.8% (3.9–31.7), 20% → 27.6% (12.7–47.2), 30% → 44.8% (26.4–64.3), and 50% → 65.5% (45.7–82.1), against 1%, 5%, 10%, 20%, 30%, and 50% expected under random selection. The confidence intervals include the random-selection benchmark at every threshold. The capture rate describes the ability of the model to rank poles at which incidents were recorded—it is not an estimate of the proportion of incidents that retrofitting those poles would prevent. AUC, area under the receiver operating characteristic curve; CI, confidence interval.

## Data Availability

Restrictions apply to the availability of these data. The outage records and the utility pole database were obtained from the Korea Electric Power Corporation (KEPCO) under data-use restrictions and are not publicly available from the authors. The environmental variables derived from them are subject to the same restrictions, and the authors are not permitted to redistribute them. Eleven Python analysis scripts and a README containing execution instructions and an input-variable dictionary are provided in the Supplementary Materials (File S1). Input-path settings are included in each script. They implement the statistical analyses described in the Methods and generate the corresponding tables and figures from analysis-ready inputs. The upstream GIS extraction, the NDVI composite, the solar index, and the photographic determinations are outside the scope of the code. Requests for the source data should be directed to KEPCO.

## Supplementary Materials

The following supporting information can be downloaded at: https://www.mdpi.com/article/10.3390/biology15211958/s1, Table S1: Snake-contact incidents, utility poles, and electrocution rate by KEPCO regional headquarters, with each region compared against the remainder of the country; Table S2: Proportion of snake-contact incidents for which the species could be identified from a photograph, by region; Table S3: Electrocution rate per 100,000 poles by region under four analysis sets, showing the effect of restricting the analysis to species-identified incidents; Table S4: Standardised logistic regression coefficients for electrocution occurrence (189 case records at 184 poles vs. 7341 control poles), with model-based and cluster-robust *p*-values; Table S5: Stability of the standardised regression coefficients across ten 1:5 subsamples of the retained control set; Table S6: Odds ratios for the square-root-transformed distance variables expressed in the original units; Table S7: Monthly frequency of incidents tested against expected counts proportional to the number of times each calendar month was observed within the extraction window; Table S8: Sensitivity model in which the six predictors departing from linearity on the logit scale (distance to the nearest pole, elevation, pole density, distance to settlements, slope, and distance to watercourses; likelihood-ratio *p* < 0.05 in each case) were entered as natural cubic splines; Table S9: Sensitivity of the model to the analysis set, the unit of analysis, and the inclusion of additional variables; Figure S1: Flow of incident records from the KEPCO register to the case–control model, showing the number of records excluded at each stage and the reason; Figure S2: A representative snake-contact incident; Figure S3: Representative photographs from which the species could be determined; Figure S4: Representative photographs from which the species could not be determined, illustrating the circumstances under which identification failed; Figure S5: Photographic basis of the three condition categories, all *Elaphe dione*; Figure S6: Distributions of the six environmental variables that were significant in the multivariable model, at case poles and at poles with no reported incident in Daegu–Gyeongbuk; Figure S7: Receiver operating characteristic curves for the training data (apparent), fivefold cross-validation, spatial block cross-validation, and external validation in Chungbuk, and capture rate across the complete Chungbuk pole inventory. File S1: Eleven Python analysis scripts and a README with execution instructions and an input-variable dictionary—input-path settings are included in each script.
