# Supplementary material for: Snake Electrocution on Power Distribution Infrastructure in South Korea: Species Composition, Spatiotemporal Patterns, and Spatial Risk Modelling
Source: Biology (Basel). 2026 Nov 1;15(21):1958. doi: 10.3390/biology15211958 (PMC13762323; doi:10.3390/biology15211958)
Supplement: Supplementary file 1 [file biology-15-01958-s001.zip › Supplementary.pdf]

Supplementary Materials

Snake Electrocution on Power Distribution Infrastructure in South Korea · biology-4636655

Table S1. Snake-contact incidents, utility poles and electrocution rate by KEPCO regional headquarters, with each region compared against the remainder of the country.

Region	Incidents	Poles	Rate per 100,000	Rest of country	Rate ratio (95% CI)	p	Sig.
Daegu–Gyeongbuk	212	761,520	27.84	6.52	4.27 (3.53–5.17)	< 0.001	***
Northern Gyeonggi	45	230,046	19.56	9.72	2.01 (1.45–2.74)	< 0.001	***
Chungbuk	32	264,036	12.12	10.11	1.20 (0.81–1.72)	0.320	ns
Gyeongnam	40	414,105	9.66	10.30	0.94 (0.66–1.30)	0.808	ns
Gwangju–Jeonnam	52	632,255	8.22	10.58	0.78 (0.57–1.04)	0.093	ns
Gangwon	20	363,160	5.51	10.66	0.52 (0.31–0.81)	0.002	**
Jeonbuk	19	390,717	4.86	10.76	0.45 (0.27–0.71)	< 0.001	***
Gyeonggi	15	414,428	3.62	10.93	0.33 (0.18–0.55)	< 0.001	***
Busan–Ulsan	5	214,243	2.33	10.64	0.22 (0.07–0.52)	< 0.001	***
Daejeon–Sejong–Chungnam	7	522,370	1.34	11.44	0.12 (0.05–0.24)	< 0.001	***
Incheon	0	160,503	0.00	10.63	0.00 (0.00–0.22)	< 0.001	***
**Total**	**447**	**4,367,383**	**10.24**	–	–	–	

Rates are computed from all 447 incidents of the register (Set A) that fall in the eleven regions for which the utility supplied a pole count. These incidents span 1 January 2024 to 4 July 2025, that is, the complete 18-month extraction window (1 January 2024 to 30 June 2025) together with three incidents recorded on 1–4 July 2025, one each in Chungbuk, Daejeon–Sejong–Chungnam and Gyeongnam; one further record carries no date and is retained because the comparison requires only the regional headquarters field. The period of the rates is therefore slightly longer than 18 months. Restricting the rates to the complete extraction window alone changes only the three regions that contain the July incidents — Chungbuk from 12.12 to 11.74, Gyeongnam from 9.66 to 9.42 and Daejeon–Sejong–Chungnam from 1.34 to 1.15 per 100,000 — and leaves the ranking of regions and the Daegu–Gyeongbuk rate ratio essentially unchanged (4.27 against 4.33). Rates are cumulative recorded incidents per 100,000 poles over that period and are not annualised. The rate ratio compares each region with all other regions combined; p-values are from exact binomial tests of the observed count against the count expected from the region's share of the national pole inventory, and confidence intervals are exact. Two incidents recorded by the Jeju regional headquarters are excluded because a pole count was not supplied for that headquarters.

Table S2. Proportion of snake-contact incidents for which the species could be identified from a photograph, by region.

Region	Incidents	Identified	Identification rate (%)	95% CI
Daegu–Gyeongbuk	212	148	69.8	63.3–75.6
Gwangju–Jeonnam	52	23	44.2	31.6–57.7
Northern Gyeonggi	45	39	86.7	73.8–93.7
Gyeongnam	40	31	77.5	62.5–87.7
Chungbuk	32	26	81.2	64.7–91.1
Gangwon	20	18	90.0	69.9–97.2
Jeonbuk	19	17	89.5	68.6–97.1
Gyeonggi	15	11	73.3	48.0–89.1
Daejeon–Sejong–Chungnam	7	5	71.4	35.9–91.8
Busan–Ulsan	5	5	100.0	56.6–100.0
Jeju	2	0	0.0	0.0–65.8
**National**	**449**	**323**	**71.9**	**67.6–75.9**

Wilson 95% confidence intervals. Homogeneity across the ten regions with at least five incidents was rejected ($\chi^2 = 35.4$, $df = 9$, $p = 5.1 \times 10^{-5}$), indicating that identification was not missing at random with respect to region.

Table S3. Electrocution rate per 100,000 poles by region under four analysis sets, showing the effect of restricting the analysis to species-identified incidents. Rates cover the same period as Table S1 and are not annualised.

Region	All records (449)	Less 7 flagged (442)	Less 9 flagged (440)	Species-identified (323)	Rank, all	Rank, identified
Daegu–Gyeongbuk	27.84	27.05	26.92	19.43	1	1
Northern Gyeonggi	19.56	19.56	19.56	16.95	2	2
Chungbuk	12.12	12.12	11.74	9.85	3	3

Region	All records (449)	Less 7 flagged (442)	Less 9 flagged (440)	Species-identified (323)	Rank, all	Rank, identified
Gyeongnam	9.66	9.66	9.66	7.49	4	4
Gwangju–Jeonnam	8.22	8.07	8.07	3.64	5	7
Gangwon	5.51	5.51	5.51	4.96	6	5
Jeonbuk	4.86	4.86	4.86	4.35	7	6
Gyeonggi	3.62	3.62	3.62	2.65	8	8
Busan–Ulsan	2.33	2.33	2.33	2.33	9	9
Daejeon–Sejong–Chungnam	1.34	1.34	1.34	0.96	10	10
Incheon	0.00	0.00	0.00	0.00	11	11

Columns are the full register, the register less the seven incidents whose field notes record a different or uncertain cause, the register less all nine such incidents, and the species-identified incidents only. These are the same exclusion criteria as Sets C, D and E in Section 2.1.1, but applied to the full register rather than to the incidents with coordinates, so the counts differ from those of the spatial analysis sets (423, 421 and 323). The ranking of the four highest-rate regions is unchanged across all four sets. Gwangju–Jeonnam, where identification was least complete (44.2%), rises from 3.64 to 8.22 per 100,000 and changes from significantly below the rest of the country ($p < 0.001$) to indistinguishable from it ($p = 0.093$).

Table S4. Standardised logistic regression coefficients for electrocution occurrence (189 case records at 184 poles vs 7,341 control poles), with model-based and cluster-robust p-values.

Variable	β	SE	OR	95% CI	p	Sig.	p (clustered)	Sig.	VIF
POL_DEN	-0.923	0.221	0.397	0.258–0.613	< 0.001	***	< 0.001	***	2.50
FF_PROX	-0.757	0.197	0.469	0.319–0.690	< 0.001	***	< 0.001	***	2.62
SET_DIST	-0.597	0.128	0.550	0.428–0.707	< 0.001	***	< 0.001	***	1.42
FOR_COV	-0.387	0.128	0.679	0.528–0.873	0.002	**	0.006	**	2.48
WAT_DIST	-0.220	0.081	0.803	0.685–0.941	0.007	**	0.011	*	1.10
ELEV	-0.132	0.096	0.876	0.725–1.058	0.170	ns	0.164	ns	1.56
SOLAR	-0.003	0.078	0.997	0.855–1.163	0.973	ns	0.969	ns	1.02
SLOPE	0.029	0.093	1.030	0.858–1.235	0.754	ns	0.704	ns	1.71
POL_DIST	0.146	0.053	1.157	1.043–1.283	0.006	**	0.023	*	1.21
NDVI	0.150	0.094	1.162	0.966–1.398	0.112	ns	0.137	ns	1.60

All variables were standardised to z-scores, so odds ratios refer to a one-standard-deviation increase. FF_PROX, SET_DIST and WAT_DIST were square-root transformed before standardisation. CIs and model-based p-values are Wald-based. Clustered p-values use standard errors clustered on a 10 km spatial grid (222 clusters); the same six variables were significant at 5 km (795 clusters) and 20 km (64 clusters). Intercept $\beta = -4.247$ (SE 0.131). AIC 1671.4. OR, odds ratio; VIF, variance inflation factor.

Table S5. Stability of the standardised regression coefficients across ten 1:5 subsamples of the retained control set.

Variable	β, full model	Mean β, ten replicates	SD	Sign agreement
FF_PROX	−0.757	−0.733	0.081	10/10
POL_DEN	−0.923	−0.944	0.112	10/10
SET_DIST	−0.597	−0.588	0.069	10/10
FOR_COV	−0.387	−0.396	0.033	10/10
WAT_DIST	−0.220	−0.249	0.047	10/10
ELEV	−0.132	−0.156	0.033	10/10
SOLAR	−0.003	−0.010	0.042	6/10
SLOPE	+0.029	+0.043	0.059	8/10
POL_DIST	+0.146	+0.202	0.010	10/10
NDVI	+0.150	+0.161	0.036	10/10

Sign agreement is the number of replicate models in which the coefficient had the same sign as the coefficient estimated from the full model. Every coefficient that was significant in the full model agreed in sign in all ten replicates; the two variables with lower agreement (SOLAR, SLOPE) have coefficients indistinguishable from zero. The per-replicate identifier lists and the random seed of the original control draw were not retained, so these ten replicates are subsamples of the retained control set rather than fresh draws from the provincial inventory, and are not a reproduction of the original draw (Section 2.2.4).

Table S6. Odds ratios for the square-root-transformed distance variables expressed in the original units.

Variable	0 → 100 m	100 → 200 m	200 → 300 m	300 → 500 m	500 → 1000 m
FF_PROX	0.510	0.757	0.808	0.713	0.536
SET_DIST	0.585	0.801	0.843	0.763	0.609
WAT_DIST	0.867	0.943	0.956	0.931	0.876

Because a square-root transformation was applied before standardisation, the odds ratio associated with a given increase in distance is not constant across the range of the variable. Values are computed from the fitted model and hold the other variables constant.

Table S7. Monthly frequency of incidents tested against expected counts proportional to the number of times each calendar month was observed within the extraction window.

Test	n	χ^2	df	p	Largest standardised residuals
Full register, twelve months	445	1216.9	11	< 0.001	June +26.8, July +11.9, May +8.0
Identified individuals, twelve months	321	931.0	11	< 0.001	June +24.2, July +8.6, May +7.4
**Full register, activity season (Apr–Oct)**	**441**	**490.6**	**6**	**< 0.001**	**June +16.0, July +6.0, May +1.9**
**Identified individuals, activity season**	**320**	**377.7**	**6**	**< 0.001**	**June +14.5, July +3.9, May +2.0**

January to June were observed twice within the extraction window (1 January 2024 – 30 June 2025) and July to December once, giving 18 month-observations rather than 19. Tests are restricted to the complete extraction window. The three incidents recorded on 1–4 July 2025 and one incident without a recorded date are excluded, leaving 445 dated incident records for the twelve-month full-register test and 321 dated identified individuals for the corresponding identified-individual test. The activity-season tests shown in bold are reported in the main text; the twelve-month tests are of limited biological interest for a seasonally inactive taxon.

Table S8. Sensitivity model in which the six predictors departing from linearity on the logit scale (distance to the nearest pole, elevation, pole density, distance to settlements, slope and distance to watercourses; likelihood-ratio $p < 0.05$ in each case) were entered as natural cubic splines.

Variable	df	LR χ^2	p	Sig.	vs linear model
FF_PROX	1	8.40	0.004	**	Retained
POL_DEN	3	27.24	< 0.001	***	Retained
SET_DIST	3	27.15	< 0.001	***	Retained
FOR_COV	1	3.33	0.068	ns	Lost
WAT_DIST	3	11.09	0.011	*	Retained
ELEV	3	20.90	< 0.001	***	Gained
SOLAR	1	0.24	0.627	ns	–
SLOPE	3	7.43	0.059	ns	–
POL_DIST	3	36.22	< 0.001	***	Retained
NDVI	1	1.48	0.224	ns	–

Pole density, distance to settlements, distance to watercourses, elevation, slope and distance to the nearest pole were entered as natural cubic splines with three degrees of freedom; the remaining variables are linear. Each row is a likelihood-ratio test of the overall effect of that variable. AIC improved from 1671.4 (linear) to 1616.8, and residual Moran's I fell from +0.051 to +0.035. The spline specification therefore improved in-sample fit and reduced, but did not eliminate, residual spatial autocorrelation. The linear model is retained as the primary specification for interpretability; the number of events per estimated coefficient falls from 18.9 to 8.6 in this model, which we treat as a guide rather than a threshold. The two specifications were not compared on validation performance.

Table S9. Sensitivity of the model to the analysis set, the unit of analysis and the inclusion of additional variables.

Model variant	Cases	FF_PROX OR	p	Significant	Max |ΔOR|
**Primary (Set B, 189 records)**	**189**	**0.469**	**< 0.001**	**6/10**	**–**
One record per case pole	184	0.468	< 0.001	6/10	0.024
Pooled control draw retaining repeat draws	189	0.470	< 0.001	6/10	0.002
Set C — 7 uncertain-cause incidents excluded	187	0.447	< 0.001	6/10	0.043
Set D — 9 uncertain-cause incidents excluded	186	0.455	< 0.001	6/10	0.043
Set E — species-identified incidents only	148	0.410	< 0.001	7/10	0.102
Northness index retained	189	0.466	< 0.001	6/10	0.050
Distance to roads added	189	0.398	< 0.001	6/10	0.248
NDVI-derived edge	189	0.487	< 0.001	5/10	0.196

Model variant	Cases	FF_PROX OR	p	Significant	Max |ΔOR|
density added					

Max |ΔOR| is the largest absolute difference in any odds ratio relative to the primary model. The odds ratio for joint proximity remained between 0.40 and 0.49 and significant at $p < 0.001$ in every variant. The primary model uses one entry per control pole; the pooled draw retaining repeat draws is shown for comparison (Section 2.2.4). The NDVI-derived edge density index is collinear with FF_PROX and its inclusion reduces the number of significant variables without improving fit.

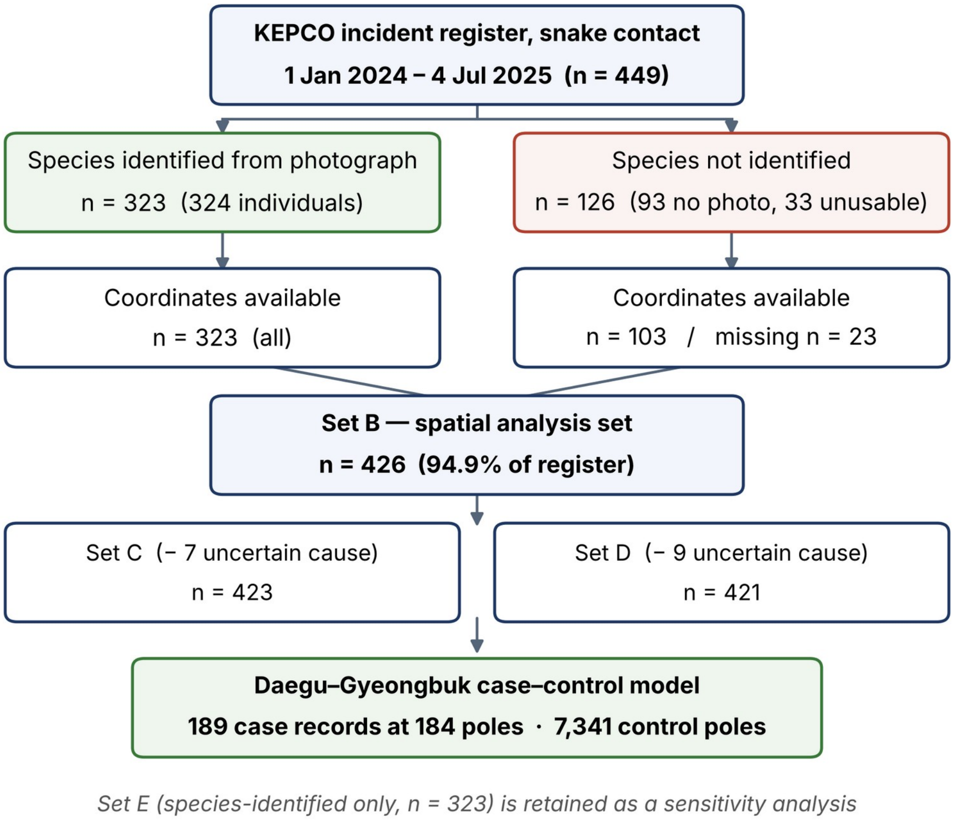

Figure S1. Flow of incident records from the KEPCO register to the case–control model, showing the number of records excluded at each stage and the reason. Set E, the species-identified subset, is retained as a sensitivity analysis.

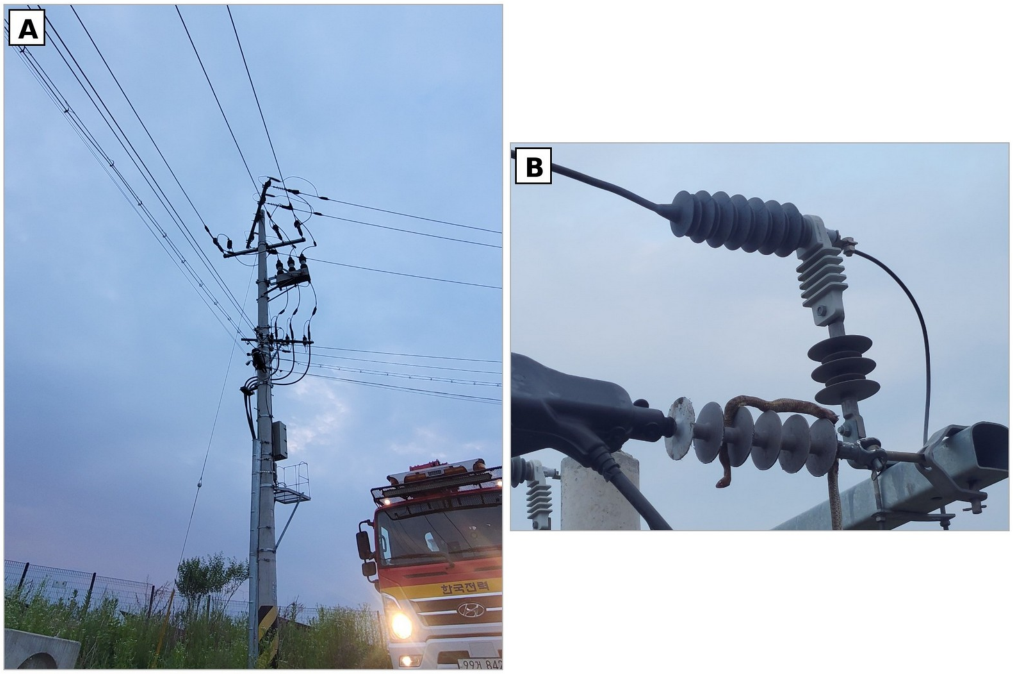

Figure S2. A representative snake-contact incident. (A) The pole at which the incident occurred, photographed by the responding field crew. (B) The animal on the energised pole-mounted equipment. Photographs of this kind are the source of the species and condition data used in this study. Pole coordinates are taken from the pole identifier in the incident record and from the utility pole database, not from the photograph (Section 2.1.1).

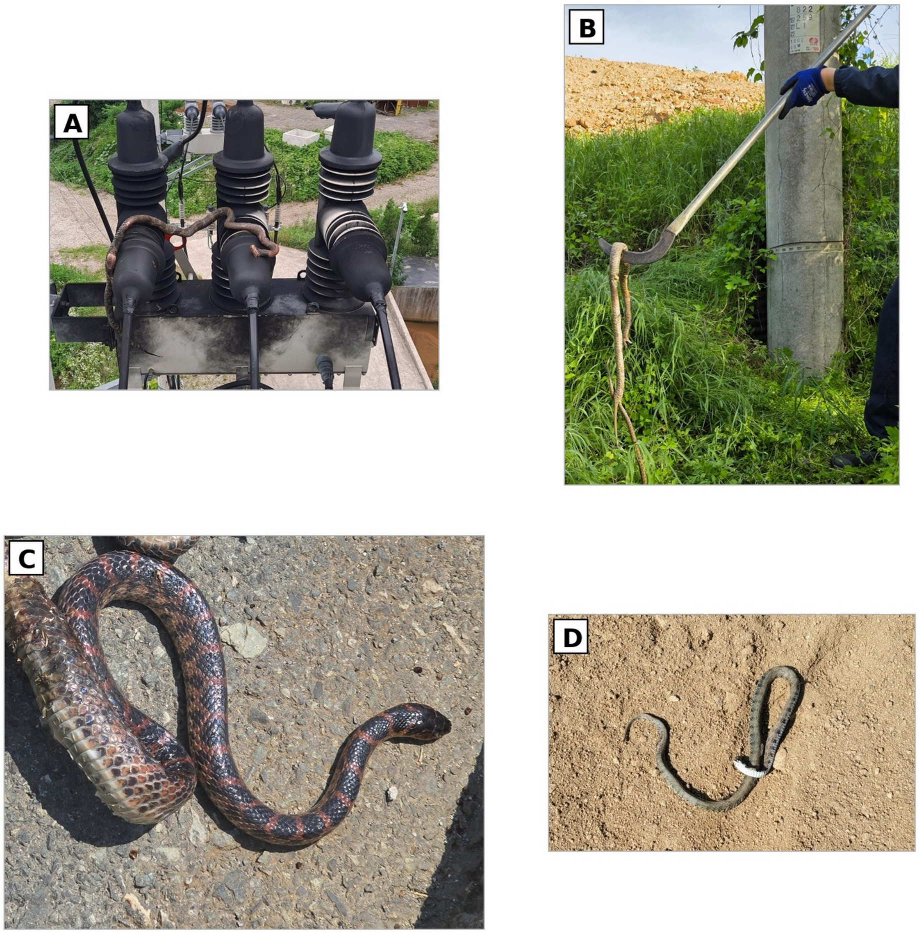

Figure S3. Representative photographs from which the species could be determined. (A) Elaphe schrenckii on pole-mounted switchgear; (B) E. dione, the two individuals recorded in the same incident, whose photographs showed genital association consistent with mating, recovered from the base of the pole; (C) Lycodon rufozonatus; (D) Rhabdophis tigrinus. Images have been cropped to the animal. Identification was based on body size, body form, and the dorsal colour pattern and the presence and arrangement of blotches or bands (Section 2.1.2).

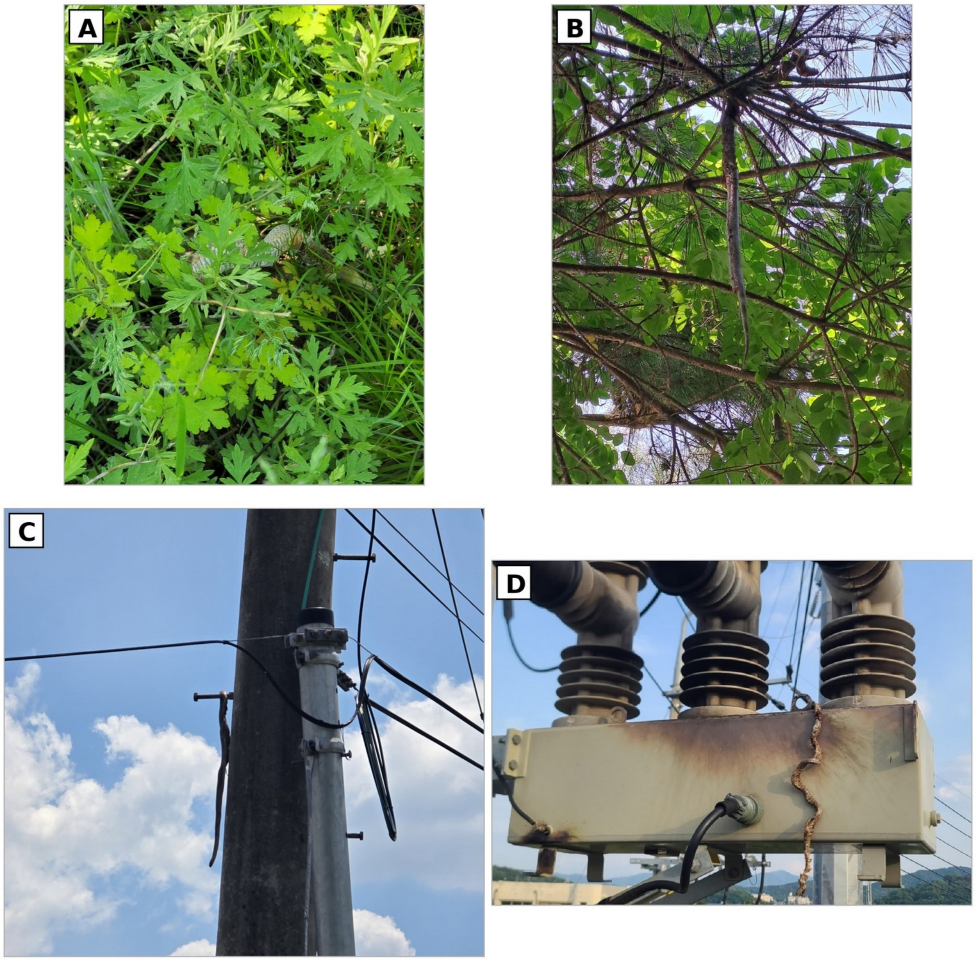

Figure S4. Representative photographs from which the species could not be determined, illustrating the circumstances under which identification failed. (A) The animal is concealed in dense ground vegetation and only a short section of the body is visible. (B) The animal is draped in the canopy above the line and visible only in silhouette against the sky. (C) The animal is on the pole at a distance at which no diagnostic character can be resolved. (D) The remains are destroyed by the fault itself. Incidents of this kind were recorded as not identifiable rather than assigned to a best guess, and account for 33 of the 126 incidents without a species determination. Annotations and crops were applied for presentation; where incidental information such as a pole identification plate is visible it is not used in the analysis.

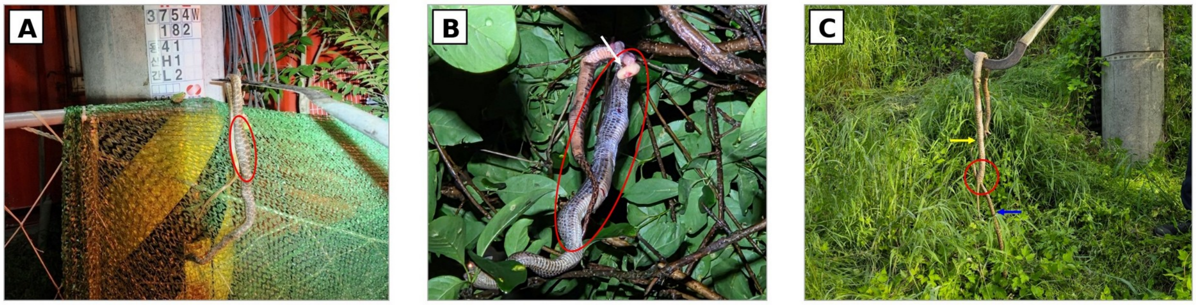

Figure S5. Photographic basis of the three condition categories, all Elaphe dione. (A) A localised swelling of part of the abdomen only, marked by the red circle, interpreted as consistent with possible recent feeding. (B) An abdomen distended evenly along its whole length, marked by the red circle, with an egg partly extruded in the photograph (white arrow): the extruded egg makes this the one individual for which gravidity is directly evidenced, whereas the distension alone was interpreted as consistent with possible gravidity. (C) Visible genital association interpreted as consistent with mating: the yellow arrow marks the female, the blue arrow the male, and the red circle the point of association. Annotations were added by the herpetologist who made the determinations (J.-H. Lee) and indicate the features on which each assessment was based. These are photographs taken after the incident: they record external appearance, are not independent confirmation of physiological state, and do not establish what the animals were doing at the moment of contact.

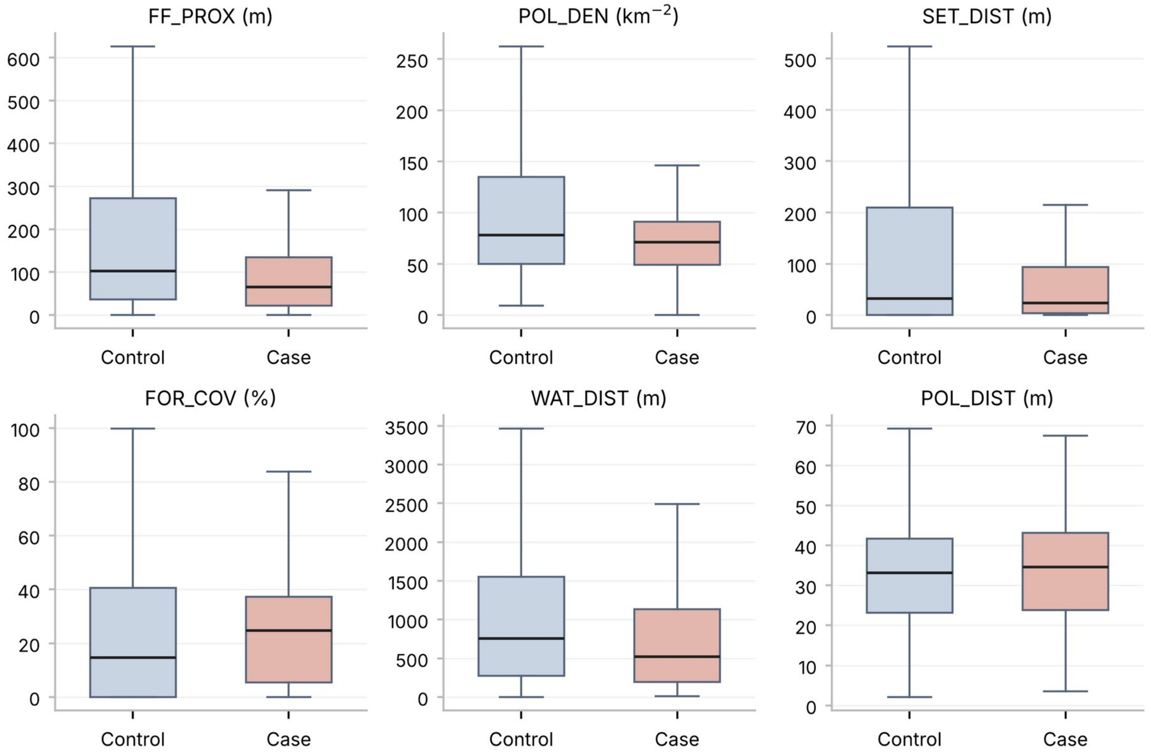

Figure S6. Distributions of the six environmental variables that were significant in the multivariable model, at case poles and at poles with no reported incident in Daegu–Gyeongbuk. Boxes show the median and interquartile range; whiskers extend to 1.5 times the interquartile range.

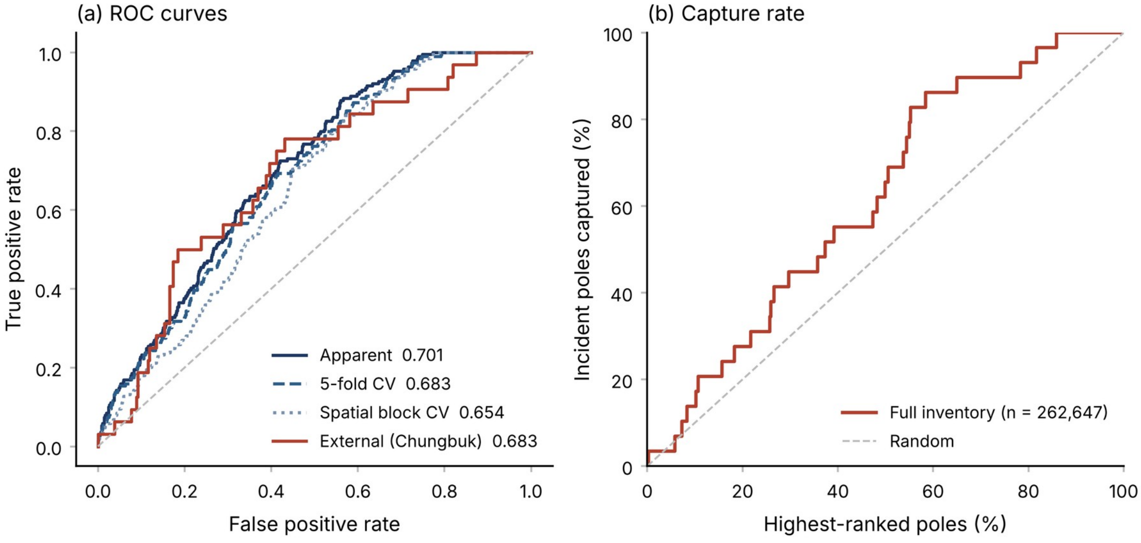

Figure S7. (a) Receiver operating characteristic curves for the training data (apparent), five-fold cross-validation, spatial block cross-validation and external validation in Chungbuk. (b) Capture rate across the complete Chungbuk pole inventory (262,647 poles, 29 matched to a recorded incident): the proportion of incident poles contained within each highest-ranked subset, against the proportion expected under random selection. Exact binomial confidence intervals for these proportions are given in Table 4; they include the random-selection benchmark at every threshold.
